# Clinical and diagnostic values of metagenomic next-generation sequencing for infection in hematology patients: a systematic review and meta-analysis

**DOI:** 10.1186/s12879-024-09073-x

**Published:** 2024-02-07

**Authors:** Yuhui Chen, Jinjin Wang, Ting Niu

**Affiliations:** Department of Hematology, West China Hospital, Sichuan University, Chengdu, China

**Keywords:** Metagenomic next-generation sequencing, Infection, Hematology patients, Diagnosis, Prognosis, Meta-analysis

## Abstract

**Objectives:**

This meta-analysis focused on systematically assessing the clinical value of mNGS for infection in hematology patients.

**Methods:**

We searched for studies that assessed the clinical value of mNGS for infection in hematology patients published in Embase, PubMed, Cochrane Library, Web of Science, and CNKI from inception to August 30, 2023. We compared the detection positive rate of pathogen for mNGS and conventional microbiological tests (CMTs). The diagnostic metrics, antibiotic adjustment rate and treatment effective rate were combined.

**Results:**

Twenty-two studies with 2325 patients were included. The positive rate of mNGS was higher than that of CMT (blood: 71.64% vs. 24.82%, *P* < 0.001; BALF: 89.86% vs. 20.78%, P < 0.001; mixed specimens: 82.02% vs. 28.12%, P < 0.001). The pooled sensitivity and specificity were 87% (95%CI: 81–91%) and 59% (95%CI: 43–72%), respectively. The reference standard/neutropenia and research type/reference standard may be sources of heterogeneity in sensitivity and specificity, respectively. The pooled antibiotic adjustment rate according to mNGS was 49.6% (95% CI: 41.8–57.4%), and the pooled effective rate was 80.9% (95% CI: 62.4–99.3%).

**Conclusion:**

mNGS has high positive detection rates in hematology patients. mNGS can guide clinical antibiotic adjustments and improve prognosis, especially in China.

**Supplementary Information:**

The online version contains supplementary material available at 10.1186/s12879-024-09073-x.

## Introduction

Hematology patients are prone to various infections due to neutropenia and immunosuppression [1]. Many infections in hematology patients are hospital-acquired and have a high rate of drug resistance, and conventional antimicrobial therapy is often ineffective [2, 3]. Infectious diseases have been one of the leading causes of death in hematology patients [4]. A prospective study in which hematology patients with febrile neutropenia received short-course (3 days) or long-course (≥9 days) antimicrobial therapy with meropenem showed that the treatment failure rates were 19% and 15% for the short and long treatment groups, respectively [5]. It is essential for the treatment of infection to identify the type of pathogen in a timely manner and adjust antibiotic therapy. However, traditional methods such as blood culture are limited by their low throughput, narrow coverage of pathogen spectrum and time consumption, which has led to the irrational use of antibiotics [6].

In recent years, metagenomic next-generation sequencing (mNGS) has rapidly emerged as a technology for the detection of pathogenic microorganisms. In comparison with other conventional diagnostic technologies, mNGS has many advantages. First, mNGS covers a wide range of pathogens, such as viruses, bacteria, fungi, and parasites, that can be detected simultaneously, as long as the sample contains detectable DNA or RNA [7]. mNGS is an unbiased sampling method that enables broad identification of known and unexpected pathogens or even the discovery of new organisms [8]. On the other hand, it takes a shorter time for mNGS to report pathogens of infection [7]. The turnaround time for mNGS from receipt of the sample to completion of data analysis varies depending on the sequencing technology, method and bioinformatics analysis method, ranging from 6 hours to 7 days (average 48 hours) [9]. mNGS has shown importance in the identification and subsequent treatment of pathogens in infectious diseases. Several studies have revealed the diagnostic advantages of mNGS over traditional pathogen detection methods [10, 11]. A meta-analysis illustrated that the diagnostic efficacy of mNGS varied depending on the sample, with a sensitivity and specificity of 90 and 86% for blood specimens, 75 and 96% for cerebrospinal fluid, and 84 and 67% for orthopedic samples, respectively [12].

Infection in hematology patients is more severe than that in most other departments because the pathogens are often opportunistic pathogens due to the use of cytotoxic chemotherapy or immunosuppressive therapy and the status of hematopoietic stem cell transplantation (HSCT). However, a systematic review and meta-analysis of the clinical value of mNGS for infection in hematology patients has not been performed. Thus, this meta-analysis focused on systematically assessing the clinical value of mNGS, including diagnostic value and impact on prognosis for infection in hematology patients.

## Method

### Study outcome

After our screening, the primary outcomes included the positivity rate of the pathogen and the diagnostic value of mNGS. The secondary outcomes were the adjustment rate of antibiotics based on mNGS and the effectiveness of treatment.

### Materials and methods

Our registration number in the PROSPERO database is CRD42022384045. We followed the Preferred Reporting Items for Systematic Evaluation and Meta-Analysis (PRISMA) reporting guidelines (2020) [13] (Table S1). Ethical approval was not needed for the study.

### Search strategy

The two reviewers (YC and JW) independently searched the Embase, PubMed, Cochrane Library, Web of Science, and China National Knowledge Infrastructure (CNKI) databases from inception to August 30, 2023. The search terms used were as follows: “next-generation sequencing” OR “metagenomic next-generation sequencing” OR “NGS” OR “mNGS” AND “hematological disorders” OR “hematology department” OR “hematologic neoplasms” OR “hematological diseases” OR “hematopoietic stem cell transplantation”) AND “infection” OR “infectious diseases” OR “infectious pathogens”. The detailed search strategy was supplemented in Table S2.

### Eligibility criteria and study selection

The two reviewers (YC and JW) independently searched and screened the literature based on the following criteria. When there were disagreements, YC, JW and TN discussed thoroughly to reach an agreement. The inclusion criteria for our study were as follows: (a) cohort, prospective, or retrospective studies that assessed the clinical value of mNGS including diagnostic value and impact on prognosis of mNGS for infection in hematology patients were included; (b) the initial data on detection positive rate or diagnostic accuracy (true positive [TP], false negative [FN], true negative [TN], and false positive [FP]) or clinical prognostic indicators were available; (c) reference standards were conventional microbiological tests (CMT) or clinical diagnosis derived from clinical signs/symptoms, laboratory tests, etc. The exclusion criteria were as follows: (a) case reports, reviews, abstracts, sessions, meta-analyses or series with fewer than 10 patients or articles in languages other than English or Chinese; and (b) studies that only included patients with viral or fungal infection.

### Quality assessment

We assessed the quality of studies included in the analysis according to the QUADAS-2 criteria using Review Manager 5.4 software [14]. This tool assesses four main aspects: method of patient selection, index test, reference standard, and the flow and timing of samples/patients throughout the study.

### Data extraction

Two authors (YC, JW) independently reviewed the records, resolving issues of disagreement through consultation and jointly deciding on the final study to be included. The data were extracted according to a preliminarily designed Microsoft Excel spreadsheet. Our research extracted: (a) positive rates of mNGS in different specimen types or in different pathogens; (b) the initial data of the diagnostic performance data (TP, FN, TN, and FP); (c) the type of research, prospective or retrospective studies; (d) country; (e) population of the patients: age less than 18 years old was defined as pediatric otherwise adult; (f) sequencing platform for mNGS and extraction of DNA or RNA; (g) reference standards including conventional microbiological tests (CMT) and clinical diagnosis derived from clinical signs/symptoms, laboratory tests, etc.; (h) whether all patients are in the hematopoietic stem cell transplant (HSCT) state; (i) whether all patients are neutropenia (patients needed to meet: absolute neutrophil count (ANC) < 0.5 × 10^9^/L in peripheral blood).

### Statistical analysis

All statistical analyses were performed using Review Manager 5.4, Stata software (version 16.0) and R software (version 4.2.2). For dichotomous variables including the positive rate of different kind of pathogen, odds ratios (ORs) were used for statistical calculations by using Review Manager. The pooled sensitivity and specificity with a 95% confidence interval (CI) of mNGS were calculated by Stata software. The area under the curve (AUC) of the summary receiver operating characteristic (SROC) curve was calculated. Subgroup analyses were performed to solve the heterogeneity. The combined single sample rates including antibiotic adjustment rates and treatment efficacy rates were calculated by R software. The funnel plot method was applied to assess publication bias. The heterogeneity among studies was assessed by the chi-square test and the I^2^ statistic (I^2^ < 25%, low heterogeneity; I^2^ 25–50%, moderate heterogeneity; I^2^ > 50%, substantial heterogeneity). When there was substantial heterogeneity (I^2^ > 50%), a random effects model was used. A fixed-effect model was used when low or moderate heterogeneity existed (I^2^ < 50%). All *P* values were based on two-sided tests. *P* < 0.05 was considered statistically significant. *P* < 0.10 was considered statistically significant for heterogeneity.

## Result

### Study characteristics

**Fig. 1:**
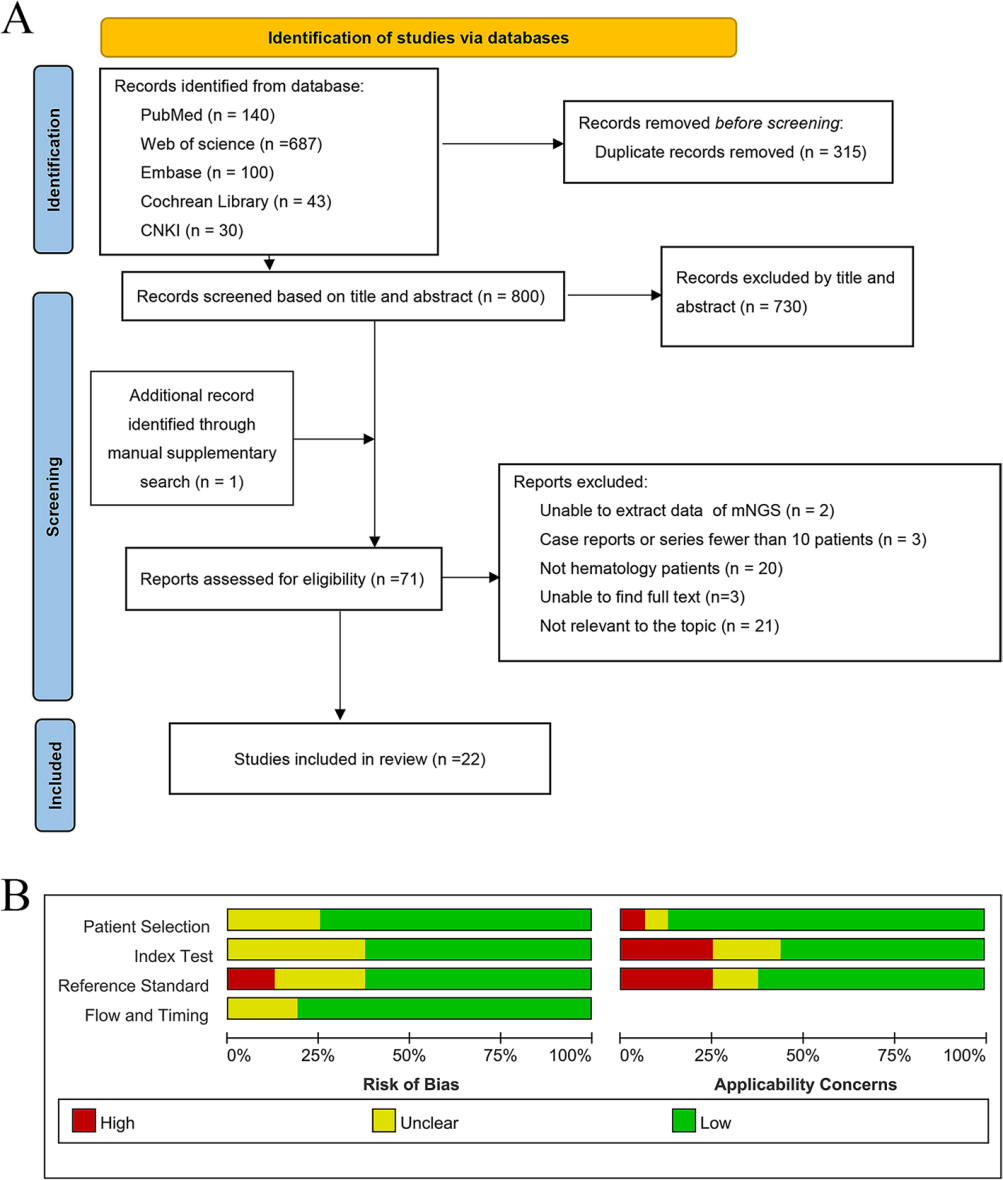
PRISMA flowchart of the study selection (**A**) and quality assessment results of included studies based on QUADAS-2 tool criteria (**B**). **A** TP = true positive; FP = false positive; TN = true negative; FN = false negative

After searching the relevant databases using the search strategy, a total of 1115 studies were screened. Of these studies, 800 were screened after removing duplicates of 315 studies. After initial screening by title and abstract, 2 articles were unable to extract mNGS data, 3 had a sample size of less than 10, 20 articles were non-hematology patients, 3 articles were unable to find the full text, and 21 articles were not relevant to the topic. Eventually, 22 studies published between 2021 and 2023 were finally included in the meta-analysis [15–36]. The sample sizes ranged from 24 to 347, with a total of 2325 patients included. The study selection process is illustrated in the PRISMA flow diagram (Fig. 1A). The characteristics of all studies are summarized in Table 1. Five (22.7%) of the included studies were prospective [15, 16, 18, 23, 32], and 17 studies were retrospective [17, 19–22, 24–35]. The vast majority (90.9%, 20/22) of studies came from China. Nine studies (40.9%) of the studies we included tested only blood specimens for mNGS [16–18, 21, 23, 29–31, 34], and the remaining study specimens included bronchoalveolar lavage fluid (BALF), cerebrospinal fluid (CSF), urine, biopsy tissue, pus, sputum and so on [15, 19, 20, 22, 24–28, 31–33, 35, 36]. Of these 22 studies, 8 (36.36%) used Illumina for sequencing [17, 19, 24–26, 29, 35, 36] and 7 (31.81%) used BGISEQ/MGISEQ for sequencing [15, 16, 20, 28, 30, 31, 33]. Fifteen (68.18%) studies extracted DNA for sequencing and 4 (18.18%) studies extracted both DNA and RNA for sequencing [15, 24, 26, 28]. Of the 22 studies, 21 (95.45%) studies reported the positive rate of mNGS and CMT for detecting pathogens, and 5 (23.80%) of them reported the respective positive detection rates for different pathogens, including bacteria, fungi, and viruses [16, 26, 28, 33, 34]. Diagnostic indicators (TP, FP, TN, TN) could be extracted from 16 (72.72%) studies [15–25, 28, 30, 32, 34, 35], of which 10 studies used clinical diagnosis as the reference standard and 6 studies used CMT as the reference standard. Fourteen (63.63%) studies reported the percentage of neutropenia in the included population and the percentage of the population treated with HSCT. There were 4 (18.18%) studies of all patients with neutropenia [16, 25, 28, 34] and 5 (22.72%) studies of all patients undergoing HSCT [15, 22, 24, 30, 32]. Eleven (50.0%) studies reported the percentage of antibiotics adjusted for mNGS [17, 19, 20, 26, 28, 29, 31–34, 36], and 4 (18.18%) of these studies further reported effective rates after adjusting for antibiotics [19, 28, 29, 36].

**Table 1:** Characteristics of the included studies

N0.	Study	Country	Type of research	Population	Sample Type	Reference Standard	Sequencing platform	DNA/RNA Extraction	Metagenomic next-generation sequencing				Neutropenia, n (%)	HSCT,n (%)
									TP	FP	FN	TN		
1	J. Yu 2021 [17]	Florida	retrospectively	Adults	Blood	Clinical Diagnosis	Illumina NexSeq500	DNA	16	5	4	7	14(45%)	20(63%)
2	W. D. Liu 2021 [16]	China	prospectively	Adults	Blood	CMT	MGISEQ-200 sequencer	DNA	14	4	2	4	14(58%)	NR
3	W. Liu 2021 [15]	China	prospectively	Adults	CSF	Clinical Diagnosis	BGISEQ-100	DNA/RNA	34	1	1	17	NR	53(100%)
4	X. Zhang 2022 [30]	China	retrospectively	Children/Adults	Blood	Clinical Diagnosis	MGISEQ-2000	DNA	31	5	3	2	23(32%)	41(100%)
5	P. Zhang 2022 [29]	China	retrospectively	Children	Blood	NR	Illumina NextseqTM	DNA	NR	NR	NR	NR	117(80%)	8(5%)
6	M. Zhang 2022 [28]	China	retrospectively	Adults	Blood; BALF; Tissue; Body fluid; Pus; Sputum	Clinical Diagnosis	BGISEQ-100	DNA/RNA	123	9	12	14	102(65%)	45(23%)
7	B. Zhang 2022 [27]	China	retrospectively	Children/Adults	BALF	NR	NR	NR	NR	NR	NR	NR	NR	NR
8	C. Xu 2022 [34]	China	retrospectively	Children/Adults	Blood	Clinical Diagnosis	NR	DNA	197	44	24	82	135(39%)	146(42%)
9	D. Wang (1) 2022 [26]	China	retrospectively	Children	BALF	NR	Illumina NextSeq550Dx	DNA/RNA	NR	NR	NR	NR	31(56%)	23(42%)
10	D. Wang (2) 2022 [25]	China	retrospectively	Children	157 Blood; 53 NPS;30 BALF; 6 Sputum; 5 Pus; 4 Hydrothorax and ascites; 3 CSF	CMT	Illumina Nextseq	DNA	70	154	8	26	194(75%)	26(10%)
11	J. H. Sun 2022 [24]	China	retrospectively	Children/Adults	75 Blood; 4 CSF; 3 Urine; 2 Pleural Fluid; 1Pus	CMT	Illumina NexSeq500	DNA/RNA	45	33	7	27	NR	112(100%)
12	E. Schulz 2022 [23]	Austria	prospectively	Adults	Blood	Clinical Diagnosis	Noscendo’s DISQVER	NR	26	5	39	26	96(100%)	43(44%)
13	Y. Qu 2022 [22]	China	retrospectively	Children	54 BALF;32 Blood; 15 CSF	Clinical Diagnosis	NR	NR	78	3	9	11	NR	101(100%)
14	S. F. Hao 2022 [21]	China	retrospectively	Children/Adults	Blood	Clinical Diagnosis	NR	DNA	57	10	25	6	NR	NR
15	F. Guo 2022 [20]	China	retrospectively	Children	49 Blood;12 BALF	CMT	BGISEQ-50/MGISEQ-2000	DNA	18	24	1	6	49(100%)	NR
16	Y. Fu 2022 [19]	China	retrospectively	Children	Blood, Urine, Sputum, CSF, BALF; Tissue	Clinical Diagnosis	Illumina NexSeq500Dx	DNA	78	2	7	9	52(54%)	NR
17	E. Benamu 2022 [18]	the United States	prospectively	Adults	Blood	Clinical Diagnosis	NR	DNA	41	0	7	7	55(100%)	NR
18	Y. Zhang 2023 [35]	China	retrospectively	Children	71 plasmas; 11 CSF;8 Sputum; 2 BALF; 1 Hydrothorax; 1 Urine; 1 Liver biopsy tissue; 1 Subcutaneous abscess puncture fluid	CMT	Illumina NexSeq500	DNA	11	44	1	40	NR	19 (28%)
19	X. Zhang 2023 [33]	China	retrospectively	Children/Adults	Blood samples; Bone marrow liquid; sputum; Pus; Nasopharyngeal/oropharyngeal/perianal swabs; Pleural effusion; Ascites, Mid-stream urine samples; Stool; Pericardial effusion	NR	BGISEQ-200	DNA	NR	NR	NR	NR	NR	28(27%)
20	Li Yuan 2023 [31]	China	retrospectively	Adults	Blood	NR	MGISEQ-2000/200	DNA	NR	NR	NR	NR	33(100%)	NR
21	S. Zhong 2023 [36]	China	retrospectively	Children	Blood; BALF; CSF; Bone marrow liquid; Pus	NR	Illumina NexSeq500	DNA	NR	NR	NR	NR	NR	NR
22	Z. Shen 2023 [32]	China	prospectively	Children/Adults	BALF	Clinical Diagnosis	NR	DNA	98	5	23	8	22 (21.78%)	134(100%)

*TP* true positive: *FP* false positive: *FN* false negative: *TN* true negative: *CMT* conventional microbiological tests: *BALF* bronchoalveolar lavage fluid: *HSCT* hematopoietic stem cell transplantation: *NR* not reported

### Study quality

The results of the quality assessment of the 16 included studies that could extract the diagnostic indicators (TP, FP, TN, TN) by the QUADAS-2 tool are presented in Fig. 1B. The majority of studies had a low risk of bias for patient selection, flow and timing. For index test bias, five studies were at an unclear risk because the information provided in the article was not sufficient to ensure that the index test was interpreted without knowing the results of the reference standard or that the specific operational procedures were not described. Regarding concerns about applicability, three studies had high concerns, and two were unclear. For the reference standard, most studies chose unclear bias because they could not determine whether the target condition could be classified. Most of the included studies had low levels of concern about applicability. More evaluation details have been shown in Table S3.

### Positive pathogen detection rate of mNGS versus CMT

**Fig. 2:**
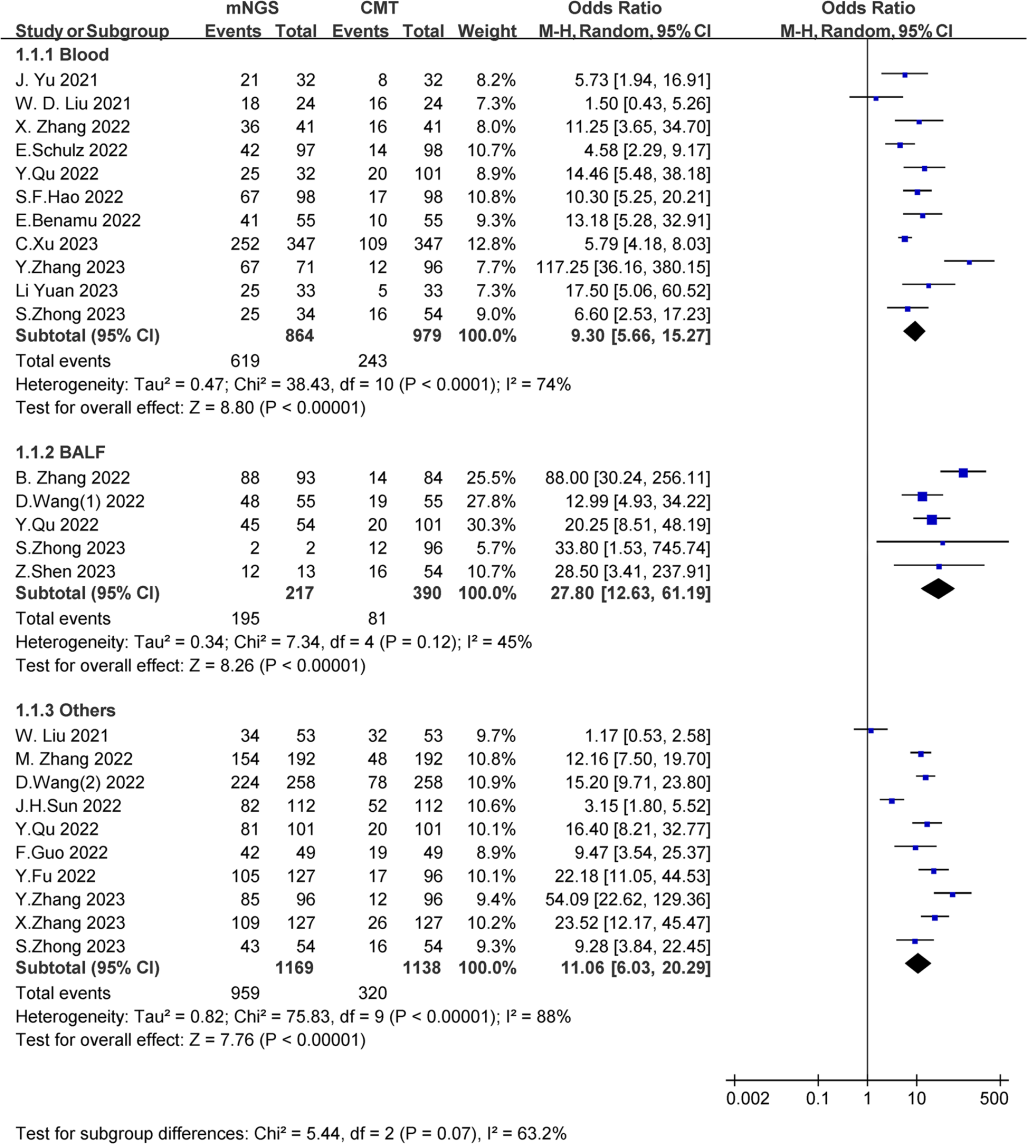
Comparison of pathogen detection positive rate between mNGS and CMT group

We calculated the pathogen detection positive rate of these 21 studies that reported the pathogen positive rate. The random effects model was used to calculate the pooled odds ratio (OR). We introduced subgroups according to different specimen types, including the blood group, BALF group, and mixed group. The overall positive rate of mNGS with blood was 71.64% (619/864), which was significantly higher than the positive rate of 24.82% (243/979) for CMT [OR = 9.30, 95% CI (5.66, 15.27), I^2^ = 74%, *P* < 0.001]. Meanwhile, the positive rate of mNGS with BALF was higher than that of CMT (89.86%, 195/217 vs. 20.78%, 81/390) [OR = 27.80, 95% CI (12.63, 61.19), I^2^ = 45%, *P* < 0.001]. For mixed specimens, the positive rate of mNGS remained higher than that of CMT (82.02,959/1169 vs. 28.12%,210/1138) [OR = 11.06, 95% CI (6.03, 20.09), I^2^ = 88%, P < 0.001] (Fig. 2). The funnel plot showed no obvious publication bias (Fig. S1). Sensitivity analyses were seen in the supplementary file (Fig. S2, Fig. S3, Fig. S4).

In addition, 745 cases reported bacterial, fungal, and viral positive rates. Our study showed that mNGS could improve the positive rate compared to CMT in the detection of bacteria, fungi and viruses (bacteria: 38.79%, 289/745 vs. 16.91%, 126/745; OR = 2.76, 95% CI (1.28, 5.96), *P* < 0.001; I^2^ = 87%) (fungi: 19.46%, 145/745 vs. 6.17%, 46/745; OR = 3.86, 95% CI (2.17, 6.66), P < 0.001; I^2^ = 50%) (virus: 51.14%, 381/745 vs. 12.21%, 91/745; OR = 6.80, 95% CI (3.76, 12.28), *P* < 0.001; I^2^ = 74%) (Fig. S5). There was no obvious publication bias (Fig. S6).

Of the four studies that reported positive rates for mNGS in the neutropenia group and non-neutropenia groups, the overall positivity rate was slightly higher in the neutropenia group than in the non- neutropenia group (79.55%, 393/494 vs. 77.98%, 255/327), but the difference was not statistically significant (OR = 0.88, 95% CI (0.45, 1.70), *P* = 0.70; I^2^ = 54%) (Fig. S7).

### Diagnostic value of mNGS

**Fig. 3:**
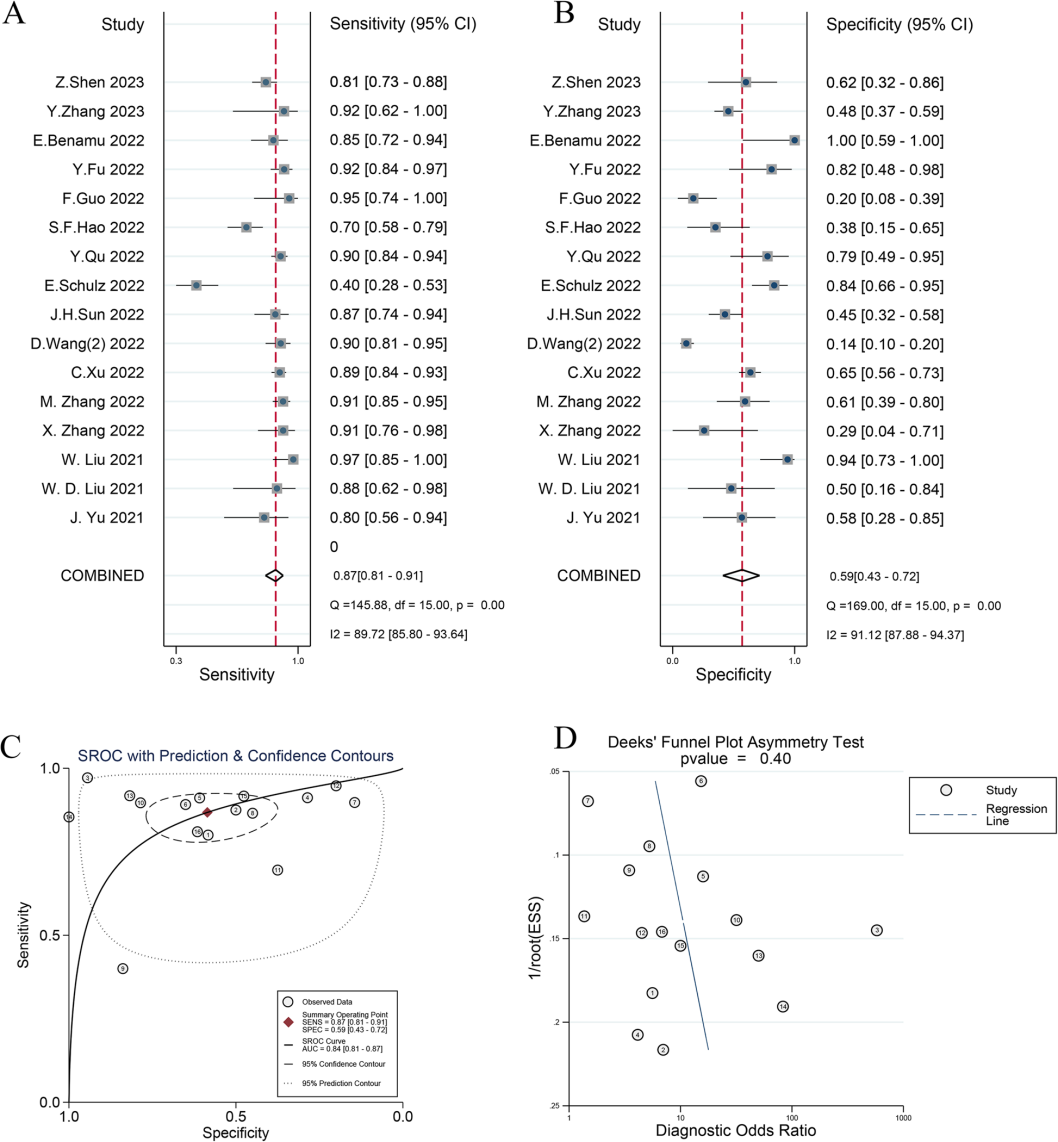
Forest plot for the sensitivity of mNGS for the diagnosis of infection in hematology patients (**A**) and forest plot for the specificity of mNGS for the diagnosis of infection in hematology patients (**B**) and the SROC curve of mNGS diagnosis of infection in hematology patients (**C**) and Deek’s Funnel Plot (**D**)

The sensitivity of mNGS for the diagnosis of infection in hematology patients ranged from 70% (95% CI: 59–88%) to 97% (95% CI: 58–79%). The specificity of mNGS for the diagnosis of infection in hematology patients ranged from 14% (95% CI: 10–20%) to 100% (95% CI: 59–100%). The pooled sensitivity and specificity were 87% (95% CI: 81–91%, I^2^ = 89.72%, *P* < 0.001) and 59% (95% CI: 43–72%, I^2^ = 91.12%, P < 0.001), respectively (Fig. 3A and B). The AUC of the SROC curve was 0.84 (95% CI: 0.81–0.87) (Fig. 3C), indicating good performance of mNGS in diagnosing infection. Deek’s test yielded no evidence of publication bias (Fig. 3D). Considering that the choice of reference standard is crucial for the calculation of diagnostic metrics, the sensitivity and specificity of mNGS were 85% (95% CI: 77–91%, I^2^ = 92.80%, *P* < 0.001) and 71% (95% CI: 57–81%, I^2^ = 66.15%, P < 0.001) when clinical diagnosis was used as the reference standard (Fig. S8), and the sensitivity and specificity of mNGS were 89% (95% CI: 83–93%, I^2^ = 0.00%, *P* = 0.52) and 32% (95% CI: 19–48%, I^2^ = 90.58%, P < 0.001) when CMT was used as the reference standard (Fig. S9), respectively.

### Subgroup analysis of diagnostic value

We conducted sensitivity and specificity subgroup analyses to search for sources of heterogeneity. Subgroup analyses were performed on the predefined subgroups for the research type, reference standard, population of patients, whether all patients had neutropenia and whether all patients had HSCT status used in the assay. If I^2^ < 50% or *P* > 0.05, heterogeneity in this subgroup was defined as low. The choice of reference standard and the presence or absence of combined neutropenia affected the sensitivity of mNGS, which was worse in the clinically diagnosed group than in the CMT group (85% (95% CI: 79–91%) vs. 91% (95% CI: 83–98%), *P* = 0.01) and worse in the neutropenia group than in the non- neutropenia group (76% (95% CI: 58–93%) vs. 89% (95% CI: 83–94%), P = 0.01). Subgroup analyses for the specificity of mNGS revealed that the research type and reference standard may be the source of heterogeneity. The different types of studies (retrospective vs. prospective) (47% (95% CI: 33–62%) vs. 82% (95% CI: 67–97%, P = 0.01) and the choice of reference standard (clinical diagnosis vs. CMT) (70% (95% CI: 59–82%) vs. 32% (95% CI: 17–48%), P = 0.01) had an effect on the specificity of mNGS for infection. The population of patients and whether all patients were in the HSCT state did not affect either the heterogeneity of sensitivity or specificity. More detailed results of the subgroup analysis are shown in Table 2.

**Table 2:** The results of subgroup analysis

Variables	Subgroup	Number of studies	Sensitivity estimate (95% CI)	P value	Specificity estimate (95% CI)	*P* value
**Research type**						
	Retrospectively	11	0.89 (0.84–0.94)	0.27	0.47 (0.33–0.62)	**0.01**
	Prospectively	5	0.81 (0.69–0.92)		0.82 (0.67–0.97)	
**Reference Standard**				**0.01**		**0.01**
	Clinical Diagnosis	11	0.85 (0.79–0.91)		0.70 (0.59–0.82)	
	CMT	5	0.91 (0.83–0.98)		0.32 (0.17–0.48)	
**Population**				0.18		0.27
	Adults	6	0.84 (0.73–0.95)		0.76 (0.65–0.88)	
	Children	5	0.85 (0.74–0.95)		0.51 (0.36–0.66)	
**Neutropenia**^**a**^				**0.01**		0.51
	Yes	3	0.76 (0.58–0.93)		0.67 (0.35–1.00)	
	No	8	0.89 (0.83–0.94)		0.51 (0.30–0.73)	
**HSCT**^**b**^				0.4		0.71
	Yes	5	0.90 (0.82–0.97)		0.65 (0.42–0.88)	
	No	5	0.84 (0.74–0.93)		0.54 (0.33–0.76)	

*P* value is for the test for subgroup differences (random-effects model)

*CI* confidence interval: *CMT* conventional microbiological tests

^a^all patients are neutropenia

^b^all patients are in hematopoietic stem cell transplant state

### Impact of mNGS on clinical treatment and prognosis

**Fig. 4:**
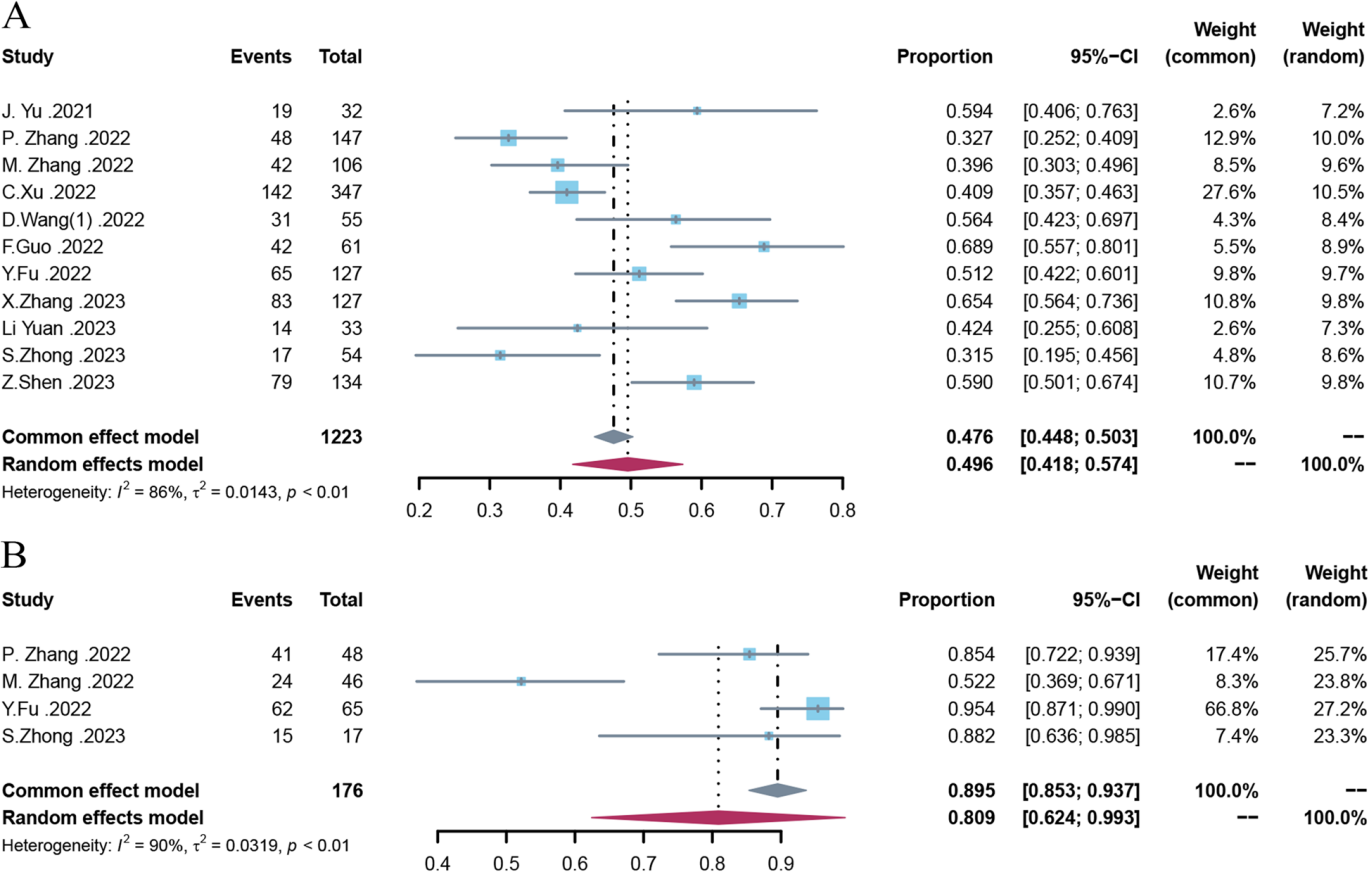
Meta-analysis of the adjustment rate of antibiotics according to mNGS (**A**); meta-analysis of the effectiveness rate after adjustment of antibiotics (**B**)

A total of 1223 cases from 11 studies reported the impact of mNGS on antibiotic adjustment. The pooled antibiotic adjustment rate according to mNGS in actual clinical practice was 49.6% (95% CI: 41.8–57.4%, I^2^ = 86%) (Fig. 4A). Four of these studies reported clinical effectiveness after antibiotic adjustment (*n* = 176), with a pooled effective rate of 80.9% (95% CI: 62.4–99.3%, I^2^ = 90%) (Fig. 4B).

## Discussion

Infection is one of the leading causes of death in hematology due to the need for large amounts of hormones, immunosuppressive drugs, broad-spectrum antibacterial drugs, chemotherapeutic drugs, etc. [37]. mNGS is widely used in hematology as an unbiased pathogen detection technique by detecting DNA or RNA [38]. The advantages of mNGS include faster, more comprehensive and more accurate data analysis, especially in the detection of specific pathogens [7, 39]. However, there are no meta-analyses assessing the practical clinical value of mNGS for infection in hematology patients. We included 22 studies to evaluate the positive detection rate, diagnostic value and clinical influence of mNGS for infection in hematology patients.

In our meta-analysis, mNGS showed a superior positive detection rate compared to CMT in both blood (71.64% vs. 24.82%, *P* < 0.001), BALF (89.86% vs. 20.78%, P < 0.001) and mixed specimens (82.02% vs. 28.12%, P < 0.001), demonstrating the advantages of mNGS for pathogen detection in a variety of specimens in hematology patients. Different specimens have different positivity rates and diagnostic value for infections. For example, pulmonary infections are one of the major causes of death in patients with hematologic malignancies, and reports have shown that approximately 30% of patients with malignancies have combined pulmonary infections [40], which can reach 70% in HSCT recipients [41]. For lower respiratory tract infections, there are many optional specimens such as transbronchial lung biopsy (TBLB), bronchoalveolar lavage fluid (BALF), and bronchial needle brushing (BB) specimens, blood and so on [42]. The Chinese expert consensus [43] published in 2023 states that mNGS is preferred for BALF in patients with lower respiratory tract infections. And blood specimens have a limited ability to detect pathogens in lower respiratory tract infections [44]. In our study, BALF has a much higher mNGS positivity rate than CMT (OR = 27.80, 95% CI (12.63, 61.19), *P* < 0.001), illustrating that BALF can be used as a preferred specimen for mNGS sent from patients with pulmonary infections. However, it’s worth mentioning that BALF specimens have their limitations and our findings may exaggerate their advantages. As a kind of open specimen, BALF is susceptible to contamination during the sampling process and can colonize nonpathogens [45]. Therefore, it is important to choose the appropriate specimen to send for testing, direct collection from the site of infection should be preferred. Whether detecting bacteria, fungi or viruses, the positive rate of mNGS was higher than that of CMT, illustrating the advantages of mNGS as a powerful complement to and extension of CMT, which helped clinicians choose targeted antibiotics. However, we cannot ignore the point that it is not uncommon for hematology patients to be tested for non-pathogenic viruses, including *EBV*, *CMV*, *HSV* or *JCV*, most of which were considered to be colonized or of no clear pathogenic significance [43]. Therefore, although the viral test positivity rate of mNGS is significantly higher than that of CMT (OR = 6.80, *P* < 0.001), its value for clinical guidance remains to be explored. All in all, considering that there is currently no unified international standard to interpreting the results of mNGS [11], the determination of mNGS reports should fully evaluate the pathogenicity, epidemiology, and bioinformatics information of the detected microorganisms, and at the same time make a comprehensive judgment based on the comprehensive combination of the clinical characteristics of the patients.

In our study, there was no difference in the rate of detection of mNGS in the neutropenia and non-neutropenia groups (79.55% vs. 77.98%, *P* = 0.70). This was inconsistent with our common viewpoint that mNGS was more advantageous in patients with neutropenia, but given that our meta-analysis only included four studies, there may be bias. The vast majority of the studies we included were premedicated with antibiotics, which have previously been reported to affect the positive of CMT. Compared to blood cultures, mNGS is less affected by widely empirical antibiotics [23, 46]. mNGS targets nucleic acid fragment sequences of pathogens that survive longer in plasma or other tissue fluids. Therefore, mNGS still has satisfactory positive rates despite the use of broad-spectrum antibiotics. All of the above findings support that mNGS remains relatively advantageous for patients with previous antibiotic exposure.

The pooled sensitivity and specificity of mNGS for infectious diseases in hematology patients were 87% (95% CI: 81–91%) and 59% (95% CI: 43–72%), respectively, indicating an excellent diagnostic performance of mNGS for infection in hematology patients. Our results are similar to the data of a retrospective study on the diagnostic performance of mNGS for infection in hematologic patients, in which the sensitivity of mNGS for pathogens was 82.6% and the specificity was 59.0% [47].

The included studies had substantial heterogeneity in sensitivity (I^2^ = 89.72%, *P* < 0.001) and specificity (I^2^ = 91.12%, P < 0.001) using a random-effects model. We further explored the sources of heterogeneity by subgroup analysis. For sensitivity, the reference standard and the presence or absence of combined neutropenia may contribute to the heterogeneity. Considering that CMT has a high negative rate, for example, the bacterial positive rate of blood cultures in patients with neutropenia is only 10–25% [48]. Therefore, the pooled sensitivity of the clinical diagnosis group was lower than that of the CMT group (85% vs. 91%, *P* = 0.01). It is worth noting that we categorized the patients into a neutropenia group and an incomplete neutropenia group based on whether all patients had neutropenia. Previous studies have reported a higher rate of positive microbiologic testing in patients with neutropenia. However, our study suggested that the sensitivity was worse in the neutropenia group (76% vs. 89%, P = 0.01). We explained that the clinical presentation of infections in patients with neutropenia is often atypical [20], and it is often difficult to determine the source of infection and select the most direct specimen for sending mNGS for testing, which can reduce the sensitivity of mNGS.

The heterogeneity of specificity was high (I^2^ = 91.12%, *P* < 0.001). First, subgroup analysis showed that research type contributes to the heterogeneity of specificity. Prospective studies can choose the proper proportion of uninfected and infected populations in their inclusion, while retrospective studies included mostly infected patients and only a minority of noninfected patients. Second, some of the studies chose CMT as the reference standard, which has a high false negative rate, leading to a low specificity [20, 21, 23–25, 49]. This was also evidenced by our subgroup analysis of the reference standard (70% vs. 32%, *P* = 0.01). Third, determining a positive threshold for mNGS in clinical applications, but there is currently no unified international standard [8]. In our included studies, there was subjectivity in the interpretation of mNGS results, and the positive reports of mNGS were mostly interpreted according to the subjective judgment of clinicians, which to some extent led to increased bias in the calculation of diagnostic metrics for mNGS.

To assess the clinical value of mNGS for patients with hematologic infections, we evaluated the pooled antibiotic adjustment rate and the pooled effectiveness rate. The antibiotic adjustment rate based on mNGS was as high as 49.6, and 80.9% of patients benefited after antibiotic adjustment. Considering that mNGS is still relatively expensive now, the rate of antibiotic adjustment based on mNGS did not seem to be very cost-effective in the studies we included (only 49.6%). We consider that hematology patients are more likely to be treated by clinicians with a wide range of broad-spectrum antibiotics at the onset of the infection, and the microorganisms detected by mNGS can often be covered by prior anti-microbial regimens. But results of mNGS still provides guidance for clinical treatment, like confirmation of the correctness of the empirical use of antibiotics. And in clinical practice, mNGS is usually a supplemental test after a failed CMT or poor treatment with the antimicrobial regimen it guides, so most patients included are those with difficult-to-obtain pathology or poor treatment, which can make it more difficult to change antibiotic regimes. More prospective studies are needed in the future to explore the optimal timing for sending mNGS.

In the real world, the application of mNGS is still a complementary diagnostic examination after the failure of traditional tests to identify the pathogen. Therefore, more prospective studies should be conducted in the future to explore the sensitivity and specificity of mNGS in the diagnosis of infection in hematology patients. On the other hand, considering that the difficulty of detecting pathogens is different for different types of infection, future studies should also explore the diagnostic value of mNGS in different types of infection, such as bloodstream infection, pulmonary infection, urinary tract infection and other infections.

Currently, mNGS is emerging as a technology that plays an important role in the detection of pathogens in infectious diseases. Our present study demonstrated the value and potential of mNGS in clinical practice in hematology patients with a high detection positive rate. Our subgroup analysis illustrated that neutropenia affects the sensitivity of mNGS. Our analysis revealed the clinical utility of mNGS for infection in hematology patients. At present, mNGS has several disadvantages, such as not being widely available in clinical practice because of its high cost and because the criteria for positivity and interpretation of mNGS are not uniform. In the future, mNGS will be an excellent tool for diagnosing infectionsin hematology patients and helping with treatment.

This study has several limitations. First, most of the studies we included were retrospective studies rather than prospective clinical controlled trials (RCTs), which can introduce bias. Second, some of the studies had relatively small sample sizes and were not convincing enough to detect the diagnostic efficacy of mNGS. Third, there are several other sources of heterogeneity in our pooled sensitivity and specificity. Because of the differences in genome length and sequencing platforms used for different types of microorganisms, it is impossible to establish a uniform positive standard for all microorganisms [50–52], so we did not perform subgroup analysis for positive criteria of mNGS. However, different positive criteria may affect the diagnostic efficacy of mNGS. In the future, updated guidelines on mNGS positive criteria for clinical practice and how to interpret mNGS results are needed.

### Supplementary Information

**Additional file 1.**

## Data Availability

The datasets generated and/or analysised during the current study are not publicly available due but are available from the corresponding author on reasonable request.

## Abbreviations

mNGS: Metagenomic next-generation sequencing
CMT: Conventional microbiological test
SROC: Summary receiver operating characteristic
HSCT: Hematopoietic stem cell transplantation
CNKI: China National Knowledge Infrastructure
TP: True positive
FN: False negative
TN: True negative
FP: False positive
ANC: Neutrophil count
AUC: Area under the curve
