# Supplementary material for: Clinical and diagnostic values of metagenomic next-generation sequencing for infection in hematology patients: a systematic review and meta-analysis
Source: BMC Infect Dis. 2024 Feb 7;24:167. doi: 10.1186/s12879-024-09073-x (PMC10848439; doi:10.1186/s12879-024-09073-x)
Supplement: Supplementary file 1 — Additional file 1. [file 12879_2024_9073_MOESM1_ESM.docx]

Supplementary Material

Clinical and diagnostic values of metagenomic next-generation sequencing for infection in hematology patients: a systematic review and meta-analysis

**Yuhui Chen^1^†, Jinjin Wang ^1^†, and Ting Niu***

†These authors contributed equally to this work and share first authorship.

^1^Department of Hematology, West China Hospital, Sichuan University, Chengdu, China

Postal address: No. 37 GuoXueXiang Street, Chengdu, Sichuan Province 610041, China.

*** Correspondence:**Ting Niu

Department of Hematology, West China Hospital, Sichuan University, Chengdu, China

Email: [niuting@wchscu.cn](mailto:niuting@wchscu.cn)

Postal address: No. 37 GuoXue Xiang, Chengdu, Sichuan Province 610041, China.

ORCID：0000-0003-1580-1014

**ADDITIONAL FILES**

**Supplementary Tables**

Table S 1 PRISMA Checklist item

Table S 2 Search strategy and results

Table S 3 Results of the evaluation of QUADAS-2 tools

**Supplementary Figures**

Figure S 1 Funnel plot of detection positive rate of different samples between mNGS and conventional methods group

Figure S2 Sensitivity analysis of plasma specimens

Figure S3 Sensitivity analysis of BALF specimens

Figure S4 Sensitivity analysis of other specimens

Figure S5 Comparison of detection positive rate of different pathogens between mNGS and CMT group

Figure S6 Funnel plot of detection positive rate of different pathogens between mNGS and conventional methods group

Figure S7 Comparison of detection positive rate of mNGS in the neutropenia group and non-neutropenia group

Figure S8 Forest plot for the sensitivity and specificity of mNGS for the diagnosis of infection in hematology patients when clinical diagnosis was used reference standard

Figure S9 Forest plot for the sensitivity and specificity of mNGS for the diagnosis of infection in hematology patients when CMT was used as reference standard

Table S 1 PRISMA Checklist item

| **Section and Topic** | **Item #** | **Checklist item** | **Location where item is reported** |
| --- | --- | --- | --- |
| **TITLE** | | |  |
| Title | 1 | Identify the report as a systematic review. | Lines 4-5 |
| **ABSTRACT** | | |  |
| Abstract | 2 | See the PRISMA 2020 for Abstracts checklist. | Lines 22-38 |
| **INTRODUCTION** | | |  |
| Rationale | 3 | Describe the rationale for the review in the context of existing knowledge. | Lines 43-69 |
| Objectives | 4 | Provide an explicit statement of the objective(s) or question(s) the review addresses. | Lines 69-73 |
| **METHODS** | | |  |
| Eligibility criteria | 5 | Specify the inclusion and exclusion criteria for the review and how studies were grouped for the syntheses. | Lines 94-102 |
| Information sources | 6 | Specify all databases, registers, websites, organisations, reference lists and other sources searched or consulted to identify studies. Specify the date when each source was last searched or consulted. | Lines 84-90 |
| Search strategy | 7 | Present the full search strategies for all databases, registers and websites, including any filters and limits used. | Table S2  Figure 1A |
| Selection process | 8 | Specify the methods used to decide whether a study met the inclusion criteria of the review, including how many reviewers screened each record and each report retrieved, whether they worked independently, and if applicable, details of automation tools used in the process. | Lines 84-85 |
| Data collection process | 9 | Specify the methods used to collect data from reports, including how many reviewers collected data from each report, whether they worked independently, any processes for obtaining or confirming data from study investigators, and if applicable, details of automation tools used in the process. | Lines 108-110 |
| Data items | 10a | List and define all outcomes for which data were sought. Specify whether all results that were compatible with each outcome domain in each study were sought (e.g. for all measures, time points, analyses), and if not, the methods used to decide which results to collect. | Lines 110-118 |
|  | 10b | List and define all other variables for which data were sought (e.g. participant and intervention characteristics, funding sources). Describe any assumptions made about any missing or unclear information. | Lines 110-118 |
| Study risk of bias assessment | 11 | Specify the methods used to assess risk of bias in the included studies, including details of the tool(s) used, how many reviewers assessed each study and whether they worked independently, and if applicable, details of automation tools used in the process. | Lines 104-106 |
| Effect measures | 12 | Specify for each outcome the effect measure(s) (e.g. risk ratio, mean difference) used in the synthesis or presentation of results. | Lines 121-127 |
| Synthesis methods | 13a | Describe the processes used to decide which studies were eligible for each synthesis (e.g. tabulating the study intervention characteristics and comparing against the planned groups for each synthesis (item #5)). | Lines 94-102 |
|  | 13b | Describe any methods required to prepare the data for presentation or synthesis, such as handling of missing summary statistics, or data conversions. | Lines 180-181 |
|  | 13c | Describe any methods used to tabulate or visually display results of individual studies and syntheses. | Lines 135-159 |
|  | 13d | Describe any methods used to synthesize results and provide a rationale for the choice(s). If meta-analysis was performed, describe the model(s), method(s) to identify the presence and extent of statistical heterogeneity, and software package(s) used. | Lines 120-121, Lines 121-126 |
|  | 13e | Describe any methods used to explore possible causes of heterogeneity among study results (e.g. subgroup analysis, meta-regression). | Lines 205-219 |
|  | 13f | Describe any sensitivity analyses conducted to assess robustness of the synthesized results. | Lines 180-181 |
| Reporting bias assessment | 14 | Describe any methods used to assess risk of bias due to missing results in a synthesis (arising from reporting biases). | Lines 182-183 |
| Certainty assessment | 15 | Describe any methods used to assess certainty (or confidence) in the body of evidence for an outcome. | Lines 130-132  Lines 122-123 |
| **RESULTS** | | |  |
| Study selection | 16a | Describe the results of the search and selection process, from the number of records identified in the search to the number of studies included in the review, ideally using a flow diagram. | Lines 135-142, Figure 1A,  Table S1 |
|  | 16b | Cite studies that might appear to meet the inclusion criteria, but which were excluded, and explain why they were excluded. | Figure 1 |
| Study characteristics | 17 | Cite each included study and present its characteristics. | Lines 140-159, Table 1 |
| Risk of bias in studies | 18 | Present assessments of risk of bias for each included study. | Lines 161-169 Table S3 |
| Results of individual studies | 19 | For all outcomes, present, for each study: (a) summary statistics for each group (where appropriate) and (b) an effect estimate and its precision (e.g. confidence/credible interval), ideally using structured tables or plots. | Table 1 |
| Results of syntheses | 20a | For each synthesis, briefly summarise the characteristics and risk of bias among contributing studies. | Lines ,170-224  Figure 2-4 |
|  | 20b | Present results of all statistical syntheses conducted. If meta-analysis was done, present for each the summary estimate and its precision (e.g. confidence/credible interval) and measures of statistical heterogeneity. If comparing groups, describe the direction of the effect. | Lines ,170-224  Figure 2-4  Figure S 2-9 |
|  | 20c | Present results of all investigations of possible causes of heterogeneity among study results. | Lines 205-219, Table 2 |
|  | 20d | Present results of all sensitivity analyses conducted to assess the robustness of the synthesized results. | Figure S2-4 |
| Reporting biases | 21 | Present assessments of risk of bias due to missing results (arising from reporting biases) for each synthesis assessed. | Lines 186-187 |
| Certainty of evidence | 22 | Present assessments of certainty (or confidence) in the body of evidence for each outcome assessed. | Lines 170-224 |
| **DISCUSSION** | | |  |
| Discussion | 23a | Provide a general interpretation of the results in the context of other evidence. | Lines 233-310 |
|  | 23b | Discuss any limitations of the evidence included in the review. | Lines 288-298 |
|  | 23c | Discuss any limitations of the review processes used. | Lines 250-252 |
|  | 23d | Discuss implications of the results for practice, policy, and future research. | Lines 311-316 |
| **OTHER INFORMATION** | | |  |
| Registration and protocol | 24a | Provide registration information for the review, including register name and registration number, or state that the review was not registered. | Lines 80-82 |
|  | 24b | Indicate where the review protocol can be accessed, or state that a protocol was not prepared. | Lines 80-81 |
|  | 24c | Describe and explain any amendments to information provided at registration or in the protocol. | none |
| Support | 25 | Describe sources of financial or non-financial support for the review, and the role of the funders or sponsors in the review. | Lines 345-349 |
| Competing interests | 26 | Declare any competing interests of review authors. | Lines 343-344 |
| Availability of data, code and other materials | 27 | Report which of the following are publicly available and where they can be found: template data collection forms; data extracted from included studies; data used for all analyses; analytic code; any other materials used in the review. | Lines 340-342 |

*From:* Page MJ, McKenzie JE, Bossuyt PM, Boutron I, Hoffmann TC, Mulrow CD, et al. The PRISMA 2020 statement: an updated guideline for reporting systematic reviews. BMJ 2021;372:n71. doi: 10.1136/bmj.n71 For more information, visit: <http://www.prisma-statement.org/>

Table S 2 Search strategy and results

| Resource | Search terms | Number |
| --- | --- | --- |
| Pubmed | ((((((((((((((((((((((((((((((((((((((((((((((((((((((((((((((((((((((((((((((((((((((((((Hematological Neoplasms[Title/Abstract]) OR (Hematological Neoplasm[Title/Abstract])) OR (Neoplasm, Hematological[Title/Abstract])) OR (Neoplasms, Hematological[Title/Abstract])) OR (Malignancies, Hematologic[Title/Abstract])) OR (Neoplasms, Hematologic[Title/Abstract])) OR (Hematologic Neoplasm[Title/Abstract])) OR (Neoplasm, Hematologic[Title/Abstract])) OR (Neoplasm, Hematologic[Title/Abstract])) OR (Hematologic Malignancies[Title/Abstract])) OR (Hematologic Malignancy[Title/Abstract])) OR (Hematological Malignancies[Title/Abstract])) OR (Hematological Malignancy[Title/Abstract])) OR (Malignancies, Hematological[Title/Abstract])) OR (Malignancy, Hematological[Title/Abstract])) OR (Malignancy, Hematologic[Title/Abstract])) OR (Hematopoietic Neoplasms[Title/Abstract])) OR (Neoplasms, Hematopoietic[Title/Abstract])) OR (Hematopoietic Neoplasm[Title/Abstract])) OR (Neoplasm, Hematopoietic[Title/Abstract])) OR (Hematopoietic Malignancies[Title/Abstract])) OR (Hematopoietic Malignancy[Title/Abstract])) OR (Malignancies, Hematopoietic[Title/Abstract])) OR (Malignancy, Hematopoietic[Title/Abstract])) OR (Lymphoma[Title/Abstract])) OR (Lymphomas[Title/Abstract])) OR (Sarcoma, Germinoblastic[Title/Abstract])) OR (Germinoblastic Sarcoma[Title/Abstract])) OR (Germinoblastic Sarcomas[Title/Abstract])) OR (Sarcomas, Germinoblastic[Title/Abstract])) OR (Reticulolymphosarcoma[Title/Abstract])) OR (Reticulolymphosarcomas[Title/Abstract])) OR (Germinoblastomas[Title/Abstract])) OR (Lymphoma, Malignant[Title/Abstract])) OR (Lymphomas, Malignant[Title/Abstract])) OR (Malignant Lymphoma[Title/Abstract])) OR (Malignant Lymphomas[Title/Abstract])) OR (Leukemia[Title/Abstract])) OR (Leukemias[Title/Abstract])) OR (Leucocythaemia[Title/Abstract])) OR (Leucocythaemias[Title/Abstract])) OR (Leucocythemia[Title/Abstract])) OR (Leucocythemias[Title/Abstract])) OR (Myeloma[Title/Abstract])) OR (Multiple Myelomas[Title/Abstract])) OR (Myelomas, Multiple[Title/Abstract])) OR (Myeloma, Plasma-Cell[Title/Abstract])) OR (Myeloma, Plasma Cell[Title/Abstract])) OR (Myelomas, Plasma-Cell[Title/Abstract])) OR (Plasma-Cell Myeloma[Title/Abstract])) OR (Plasma-Cell Myelomas[Title/Abstract])) OR (Myeloma-Multiple[Title/Abstract])) OR (Myeloma Multiple[Title/Abstract])) OR (Myeloma-Multiples[Title/Abstract])) OR (Kahler Disease[Title/Abstract])) OR (Disease, Kahler[Title/Abstract])) OR (Plasma Cell Myeloma[Title/Abstract])) OR (Cell Myeloma, Plasma[Title/Abstract])) OR (Cell Myelomas, Plasma[Title/Abstract])) OR (Myelomas, Plasma Cell[Title/Abstract])) OR (Plasma Cell Myelomas[Title/Abstract])) OR (Myeloma, Multiple[Title/Abstract])) OR (Myelomatosis[Title/Abstract])) OR (Myelomatoses[Title/Abstract])) OR (Myelomatoses[Title/Abstract]))) OR (Hematologic Diseases[Title/Abstract])) OR (Disease, Hematologic[Title/Abstract])) OR (Diseases, Hematologic[Title/Abstract])) OR (Hematologic Disease[Title/Abstract])) OR (Blood Diseases[Title/Abstract])) OR (Blood Disease[Title/Abstract])) OR (Disease, Blood[Title/Abstract])) OR (Diseases, Blood[Title/Abstract])) OR (Hematological Diseases[Title/Abstract])) OR (Disease, Hematological[Title/Abstract])) OR (Diseases, Hematological[Title/Abstract])) OR (Hematological Disease[Title/Abstract]))) OR (hematopoietic stem cell transplantation[Title/Abstract])) OR (Stem Cell Transplantation, Hematopoietic[Title/Abstract])) OR (Transplantation, Hematopoietic Stem Cell[Title/Abstract])) OR (HSCT[Title/Abstract])) OR (Peripheral Blood Stem Cell Transplantation[Title/Abstract])) OR (Stem Cell Transplantation[Title/Abstract])) OR (Relapsed[Title/Abstract] OR refractory cancer[Title/Abstract])) OR (hematological disorders[Title/Abstract])) OR (allogeneic hematopoietic stem cell transplantation)) OR (hematology department[Title/Abstract])) OR ("Hematologic Neoplasms"[Mesh])) AND ((((((((((((mNGS[Title/Abstract]) OR (Metagenomic Next- Generation Sequencing Test[Title/Abstract])) OR (plasma cell-free DNA[Title/Abstract])) OR (Metagenomics[Title/Abstract])) OR (metagenomic next-generation sequencing[Title/Abstract])) OR (High Throughput Nucleotide Sequencing Sequencing, Next-Generation[Title/Abstract])) OR (IIlumina Sequencing[Title/Abstract])) OR (Ion Torrent Sequencing[Title/Abstract])) OR (Plasma Microbial Cell-Free DNA Sequencing[Title/Abstract])) OR (Cell-Free DNA-Based Next-Generation Sequencing[Title/Abstract])) OR (Rapid Next-Generation Sequencing[Title/Abstract])) OR (Next-Generation Sequencing Technology[Title/Abstract]))) AND (((((((((Infection[Title/Abstract] OR Infestation[Title/Abstract]) OR (Infestation[Title/Abstract] OR Infection[Title/Abstract])) OR (Infections[Title/Abstract] OR Infestations[Title/Abstract])) OR (Infestations[Title/Abstract] OR Infections[Title/Abstract])) OR (Infection[Title/Abstract])) OR (infectious diseases[Title/Abstract])) OR (pathogens[Title/Abstract])) OR (infectious pathogens[Title/Abstract])) OR ("Infections"[Mesh])) | 140 |
| Web of Science | (AB=(Hematological Neoplasms OR Hematological Neoplasm OR Neoplasm, Hematological OR Neoplasms, Hematological OR Malignancies, Hematologic OR Neoplasms, Hematologic OR Hematologic Neoplasm OR Neoplasm, Hematologic OR Neoplasm, Hematologic OR Hematologic Malignancies OR Hematologic Malignancy OR Hematological Malignancies OR Hematological Malignancy OR Malignancies, Hematological OR Malignancy, Hematological OR Malignancy, Hematologic OR Hematopoietic Neoplasms OR Neoplasms, Hematopoietic OR Hematopoietic Neoplasm OR Neoplasm, Hematopoietic OR Hematopoietic Malignancies OR Hematopoietic Malignancy OR Malignancies, Hematopoietic OR Malignancy, Hematopoietic OR Lymphoma OR Lymphomas OR Sarcoma, Germinoblastic OR Germinoblastic Sarcoma OR Germinoblastic Sarcomas OR Sarcomas, Germinoblastic OR Reticulolymphosarcoma OR Reticulolymphosarcomas OR Germinoblastomas OR Lymphoma, Malignant OR Lymphomas, Malignant OR Malignant Lymphoma OR Malignant Lymphomas OR Leukemia OR Leukemias OR Leucocythaemia OR Leucocythaemias OR Leucocythemia OR Leucocythemias OR Myeloma OR Multiple Myelomas OR Myelomas, Multiple OR Myeloma, Plasma-Cell OR Myeloma, Plasma Cell OR Myelomas, Plasma-Cell OR Plasma-Cell Myeloma OR Plasma-Cell Myelomas OR Myeloma-Multiple OR Myeloma Multiple OR Myeloma-Multiples OR Kahler Disease OR Disease, Kahler OR Plasma Cell Myeloma OR Cell Myeloma, Plasma OR Cell Myelomas, Plasma OR Myelomas, Plasma Cell OR Plasma Cell Myelomas OR Myeloma, Multiple OR Myelomatosis OR Myelomatoses OR Myelomatoses OR Hematologic Diseases OR Disease, Hematologic OR Diseases, Hematologic OR Hematologic Disease OR Blood Diseases OR Blood Disease OR Disease, Blood OR Diseases, Blood OR Hematological Diseases OR Disease, Hematological OR Diseases, Hematological OR Hematological Disease OR hematopoietic stem cell transplantation OR Stem Cell Transplantation, Hematopoietic OR Transplantation, Hematopoietic Stem Cell OR HSCT OR Peripheral Blood Stem Cell Transplantation OR Stem Cell Transplantation OR Relapsed OR refractory cancer OR hematological disorders OR allogeneic hematopoietic stem cell transplantation OR hematology department) OR TS= ("Hematologic Neoplasms")) AND (AB=(mNGS OR Metagenomic Next-Generation Sequencing Test OR plasma cell-free DNA OR Metagenomics OR metagenomic next-generation sequencing OR High Throughput Nucleotide Sequencing Sequencing, Next-Generation OR IIlumina Sequencing OR Ion Torrent Sequencing OR Plasma Microbial Cell-Free DNA Sequencing OR Cell-Free DNA-Based Next-Generation Sequencing OR Rapid Next-Generation Sequencing OR Next-Generation Sequencing Technology)) AND (AB=(Infection OR Infestation OR Infestation OR Infection OR Infections OR Infestations OR Infestations OR Infections OR Infection OR infectious diseases OR pathogens OR infectious pathogens) OR TS=(Infections)) | 687 |
| Embase | #4. #1 AND #2 AND #3 100  #3. infestation:ab,ti OR infestations:ab,ti OR 2,344,348 infection:ab,ti OR 'infectious diseases':ab,ti OR pathogens:ab,ti OR 'infectious pathogens':ab,ti OR infections:ab,ti  #2. mngs:ab,ti OR 'metagenomic next-generation 12,755 sequencing test':ab,ti OR 'plasma cell-free dna':ab,ti OR metagenomics:ab,ti OR 'metagenomic next-generation sequencing':ab,ti OR 'high throughput nucleotide sequencing sequencing, next-generation':ab,ti OR 'iilumina sequencing':ab,ti OR 'ion torrent sequencing':ab,ti OR 'plasma microbial cell-free dna sequencing':ab,ti OR 'cell-free dna-based next-generation sequencing':ab,ti OR 'rapid next-generation sequencing':ab,ti OR 'next-generation sequencing technology':ab,ti  #1. 'hematological neoplasms':ab,ti OR 'hematological 798,449 neoplasm':ab,ti OR 'neoplasm, hematological':ab,ti OR 'neoplasms, hematological':ab,ti OR 'malignancies, hematologic':ab,ti OR 'neoplasms, hematologic':ab,ti OR 'hematologic neoplasm':ab,ti OR 'neoplasm, hematologic':ab,ti OR 'hematologic malignancies':ab,ti OR 'hematologic malignancy':ab,ti OR 'hematological malignancies':ab,ti OR 'hematological malignancy':ab,ti OR 'malignancies, hematological':ab,ti OR 'malignancy, hematological':ab,ti OR 'malignancy, hematologic':ab,ti OR 'hematopoietic neoplasms':ab,ti OR 'neoplasms, hematopoietic':ab,ti OR 'hematopoietic neoplasm':ab,ti OR 'neoplasm, hematopoietic':ab,ti OR 'hematopoietic malignancies':ab,ti OR 'hematopoietic malignancy':ab,ti OR 'malignancies, hematopoietic':ab,ti OR 'malignancy, hematopoietic':ab,ti OR lymphoma:ab,ti OR lymphomas:ab,ti OR 'sarcoma, germinoblastic':ab,ti OR 'germinoblastic sarcoma':ab,ti OR 'germinoblastic sarcomas':ab,ti OR 'sarcomas, germinoblastic':ab,ti OR reticulolymphosarcoma:ab,ti OR reticulolymphosarcomas:ab,ti OR germinoblastomas:ab,ti OR 'lymphoma, malignant':ab,ti OR 'lymphomas, malignant':ab,ti OR 'malignant lymphoma':ab,ti OR 'malignant lymphomas':ab,ti OR leukemia:ab,ti OR leukemias:ab,ti OR leucocythaemia:ab,ti OR leucocythaemias:ab,ti OR leucocythemia:ab,ti OR leucocythemias:ab,ti OR myeloma:ab,ti OR 'multiple myelomas':ab,ti OR 'myelomas, multiple':ab,ti OR 'myeloma, plasma-cell':ab,ti OR 'myeloma, plasma cell':ab,ti OR 'myelomas, plasma-cell':ab,ti OR 'plasma-cell myeloma':ab,ti OR 'plasma-cell myelomas':ab,ti OR 'myeloma multiple':ab,ti OR 'myeloma multiples':ab,ti OR 'kahler disease':ab,ti OR 'disease, kahler':ab,ti OR 'plasma cell myeloma':ab,ti OR 'cell myeloma, plasma':ab,ti OR 'cell myelomas, plasma':ab,ti OR 'myelomas, plasma cell':ab,ti OR 'plasma cell myelomas':ab,ti OR 'myeloma, multiple':ab,ti OR myelomatosis:ab,ti OR myelomatoses:ab,ti OR 'hematologic diseases':ab,ti OR 'disease, hematologic':ab,ti OR 'diseases, hematologic':ab,ti OR 'hematologic disease':ab,ti OR 'blood diseases':ab,ti OR 'blood disease':ab,ti OR 'disease, blood':ab,ti OR 'diseases, blood':ab,ti OR 'hematological diseases':ab,ti OR 'disease, hematological':ab,ti OR 'diseases, hematological':ab,ti OR 'hematological disease':ab,ti OR 'hematopoietic stem cell transplantation':ab,ti OR 'stem cell transplantation, hematopoietic':ab,ti OR 'transplantation, hematopoietic stem cell':ab,ti OR hsct:ab,ti OR 'peripheral blood stem cell transplantation':ab,ti OR 'stem cell transplantation':ab,ti OR relapsed:ab,ti OR 'refractory cancer':ab,ti OR 'hematological disorders':ab,ti OR 'allogeneic hematopoietic stem cell transplantation':ab,ti OR 'hematology department':ab,ti | 100 |
| Cochrean Library | #1 MeSH descriptor: [Hematologic Neoplasms] explode all trees 656  #2 (Hematological Neoplasms OR Hematological Neoplasm OR Neoplasm, Hematological OR Neoplasms, Hematological OR Malignancies, Hematologic OR Neoplasms, Hematologic OR Hematologic Neoplasm OR Neoplasm, Hematologic OR Neoplasm, Hematologic OR Hematologic Malignancies OR Hematologic Malignancy OR Hematological Malignancies OR Hematological Malignancy OR Malignancies, Hematological OR Malignancy, Hematological OR Malignancy, Hematologic OR Hematopoietic Neoplasms OR Neoplasms, Hematopoietic OR Hematopoietic Neoplasm OR Neoplasm, Hematopoietic OR Hematopoietic Malignancies OR Hematopoietic Malignancy OR Malignancies, Hematopoietic OR Malignancy, Hematopoietic OR Lymphoma OR Lymphomas OR Sarcoma, Germinoblastic OR Germinoblastic Sarcoma OR Germinoblastic Sarcomas OR Sarcomas, Germinoblastic OR Reticulolymphosarcoma OR Reticulolymphosarcomas OR Germinoblastomas OR Lymphoma, Malignant OR Lymphomas, Malignant OR Malignant Lymphoma OR Malignant Lymphomas OR Leukemia OR Leukemias OR Leucocythaemia OR Leucocythaemias OR Leucocythemia OR Leucocythemias OR Myeloma OR Multiple Myelomas OR Myelomas, Multiple OR Myeloma, Plasma-Cell OR Myeloma, Plasma Cell OR Myelomas, Plasma-Cell OR Plasma-Cell Myeloma OR Plasma-Cell Myelomas OR Myeloma-Multiple OR Myeloma Multiple OR Myeloma-Multiples OR Kahler Disease OR Disease, Kahler OR Plasma Cell Myeloma OR Cell Myeloma, Plasma OR Cell Myelomas, Plasma OR Myelomas, Plasma Cell OR Plasma Cell Myelomas OR Myeloma, Multiple OR Myelomatosis OR Myelomatoses OR Myelomatoses OR Hematologic Diseases OR Disease, Hematologic OR Diseases, Hematologic OR Hematologic Disease OR Blood Diseases OR Blood Disease OR Disease, Blood OR Diseases, Blood OR Hematological Diseases OR Disease, Hematological OR Diseases, Hematological OR Hematological Disease OR hematopoietic stem cell transplantation OR Stem Cell Transplantation, Hematopoietic OR Transplantation, Hematopoietic Stem Cell OR HSCT OR Peripheral Blood Stem Cell Transplantation OR Stem Cell Transplantation OR Relapsed OR refractory cancer OR hematological disorders OR allogeneic hematopoietic stem cell transplantation OR hematology department):ti,ab,kw (Word variations have been searched) 200934  #3 #1 or #2 201015  #4 MeSH descriptor: [Infections] explode all trees 83432  #5 (Infection OR Infestation OR Infestation OR Infection OR Infections OR Infestations OR Infestations OR Infections OR Infection OR infectious diseases OR pathogens OR infectious pathogens):ti,ab,kw (Word variations have been searched) 150494  #6 #4 or #5 181100  #7 (mNGS OR “Metagenomic Next-Generation Sequencing Test” OR “plasma cell-free DNA” OR Metagenomics OR “metagenomic next-generation sequencing” OR “High Throughput Nucleotide Sequencing Sequencing, Next-Generation” OR “IIlumina Sequencing” OR “Ion Torrent Sequencing” OR “Plasma Microbial Cell-Free DNA Sequencing” OR “Cell-Free DNA-Based Next-Generation Sequencing” OR “Rapid Next-Generation Sequencing” OR “Next-Generation Sequencing Technology”:ti,ab,kw (Word variations have been searched) 1910  #8 MeSH descriptor: [High-Throughput Nucleotide Sequencing] explode all trees 97  #9 #8 or #7 2003  #10 #3 and #6 and #9 43 | 43 |
| China National Knowledge Infrastructure (CNKI) | (Topic: Hematologic Tumors (exact) ) AND (Title: Hematologic Tumors + myelodysplastic syndromes + Hematologic Malignancies + chronic myelogenous leukemia + Hodgkin's Lymphoma + Leukemia + Hematologic Diseases + Hematologic Diseases + Oncologic Diseases + Malignant Neoplastic Diseases (exact) ) AND (Topic: Plasma Microbial Cell-Free DNA Sequencing (exact) ) OR (Title: Rapid Next-Generation Sequencing + Cell-Free DNA-Based Next-Generation Sequencing + mNGS (exact) ) ) | 30 |

**QUADAS-2 tools**

**Domain 1: Patient Selection**

Q 1: Was a consecutive or random sample of patients enrolled?

Q 2: Was a case–control design avoided?

Q 3: Did the study avoid inappropriate exclusions?

*Risk of Bias: Could the Selection of Patients Have Introduced Bias?*

*Applicability: Are There Concerns That the Included Patients and Setting Do Not Match the Review Q?*

**Domain 2: Index Test**

Q 1: Were the index test results interpreted without knowledge of the results of the reference standard?

Q 2: If a threshold was used, was it prespecified?

*Risk of Bias: Could the Conduct or Interpretation of the Index Test Have Introduced Bias?*

*Applicability: Are There Concerns That the Index Test, Its Conduct, or Its Interpretation Differ From the Review Q?*

**Domain 3: Reference Standard**

Q 1: Is the reference standard likely to correctly classify the target condition?

Q 2: Were the reference standard results interpreted without knowledge of the results of the index test?

*Risk of Bias: Could the Reference Standard, Its Conduct, or Its Interpretation Have Introduced Bias?*

*Applicability: Are There Concerns That the Target Condition as Defined by the Reference Standard Does Not Match the Q?*

**Domain 4: Flow and Timing**

Q 1: Was there an appropriate interval between the index test and reference standard?

Q 2: Did all patients receive the same reference standard?

Q 3: Were all patients included in the analysis?

*Risk of Bias: Could the Patient Flow Have Introduced Bias?*

Table S3 Results of the evaluation of QUADAS-2 tools

| Study | Patient Selection | | | | | Index Test | | | | Reference Standard | | | | Flow and Timing | | | |
| --- | --- | --- | --- | --- | --- | --- | --- | --- | --- | --- | --- | --- | --- | --- | --- | --- | --- |
|  | Q1 | Q2 | Q3 | Risk of bias | Applicability | Q1 | Q2 | Risk of bias | Applicability | Q1 | Q2 | Risk of bias | Applicability | Q1 | Q2 | Q3 | Risk of bias |
| J. Yu 2021 | yes | yes | unclear | unclear | unclear | unclear | yes | unclear | unclear | yes | no | unclear | unclear | unclear | yes | yes | unclear |
| W. D. Liu 2021 | yes | yes | unclear | low | low | yes | yes | low | unclear | unclear | yes | unclear | low | unclear | yes | yes | unclear |
| W. Liu 2021 | yes | yes | unclear | unclear | unclear | unclear | yes | unclear | low | yes | no | low | low | unclear | yes | yes | unclear |
| X. Zhang 2022 | yes | yes | unclear | low | low | yes | yes | low | low | yes | no | low | unclear | yes | yes | yes | low |
| M.Zhang 2022 | yes | yes | unclear | low | low | yes | no | low | unclear | yes | no | low | unclear | yes | yes | yes | low |
| C.Xu 2022 | yes | yes | yes | low | low | yes | no | low | low | yes | no | low | low | unclear | yes | yes | low |
| D.Wang(2) 2022 | yes | yes | yes | unclear | low | yes | no | low | low | no | yes | unclear | high | yes | no | yes | unclear |
| J.H.Sun 2022 | yes | yes | yes | low | low | unclaer | no | unclear | high | unclaer | unclear | high | high | yes | yes | yes | low |
| E.Schulz 2022 | yes | yes | unclear | low | low | yes | no | low | low | yes | unclear | unclear | low | yes | yes | yes | low |
| Y.Qu 2022 | yes | yes | unclear | low | low | no | no | unclear | high | no | yes | low | low | unclear | yes | yes | low |
| S.F.Hao 2022 | yes | yes | unclear | unclear | low | unclear | no | unclear | unclear | yes | no | low | low | yes | yes | yes | low |
| F.Guo 2022 | yes | yes | yes | low | low | yes | no | low | low | unclaer | no | unclaer | high | yes | yes | yes | low |
| Y.Fu 2022 | yes | yes | yes | low | low | yes | no | low | low | yes | no | low | low | unclear | yes | yes | low |
| E.Benamu 2022 | unclaer | yes | unclear | unclaer | high | unclear | no | unclaer | high | yes | no | low | low | yes | yes | yes | low |
| Y.Zhang 2023 | yes | yes | yes | low | low | yes | no | low | low | no | yes | high | high | yes | yes | yes | low |
| Z.Shen 2023 | yes | yes | yes | low | low | yes | no | low | low | yes | no | low | low | yes | yes | yes | low |

**Supplementary Figures**

Figure S 1 Funnel plot of detection positive rate of different samples between mNGS and conventional methods group


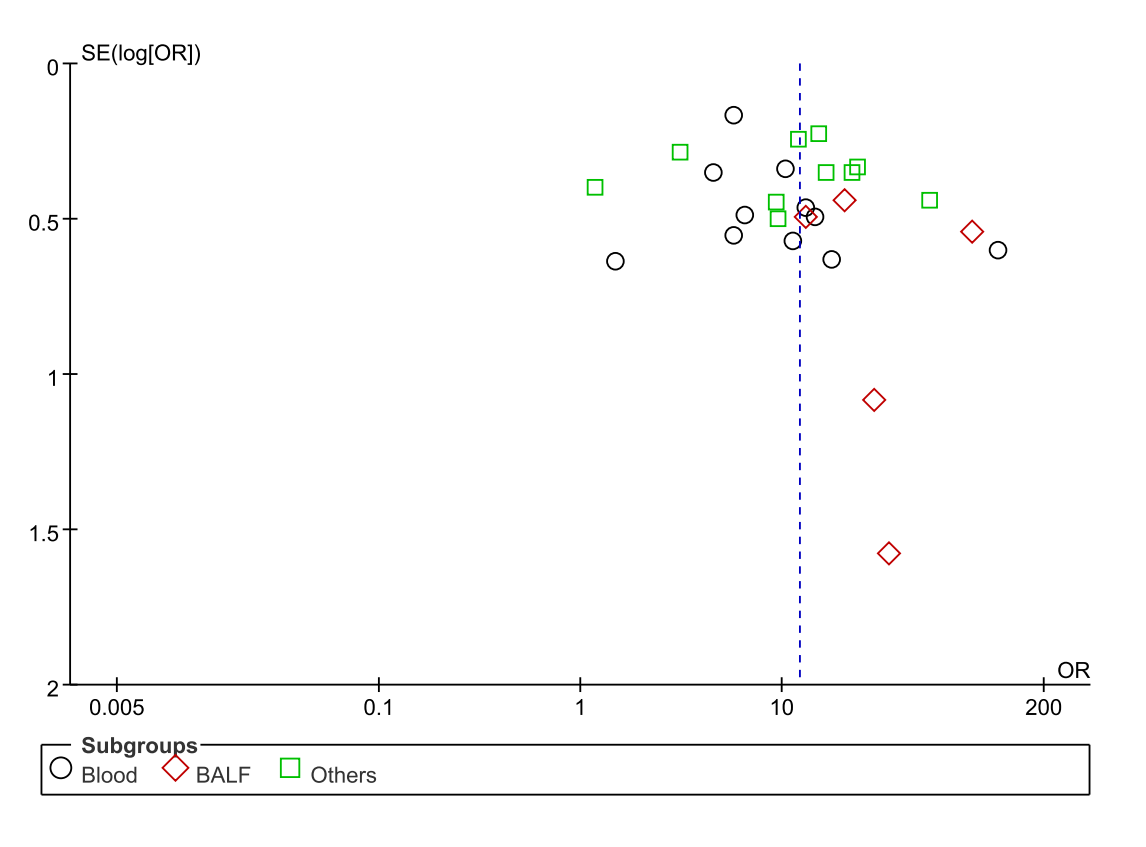


Figure S 2 Sensitivity analysis of plasma specimens


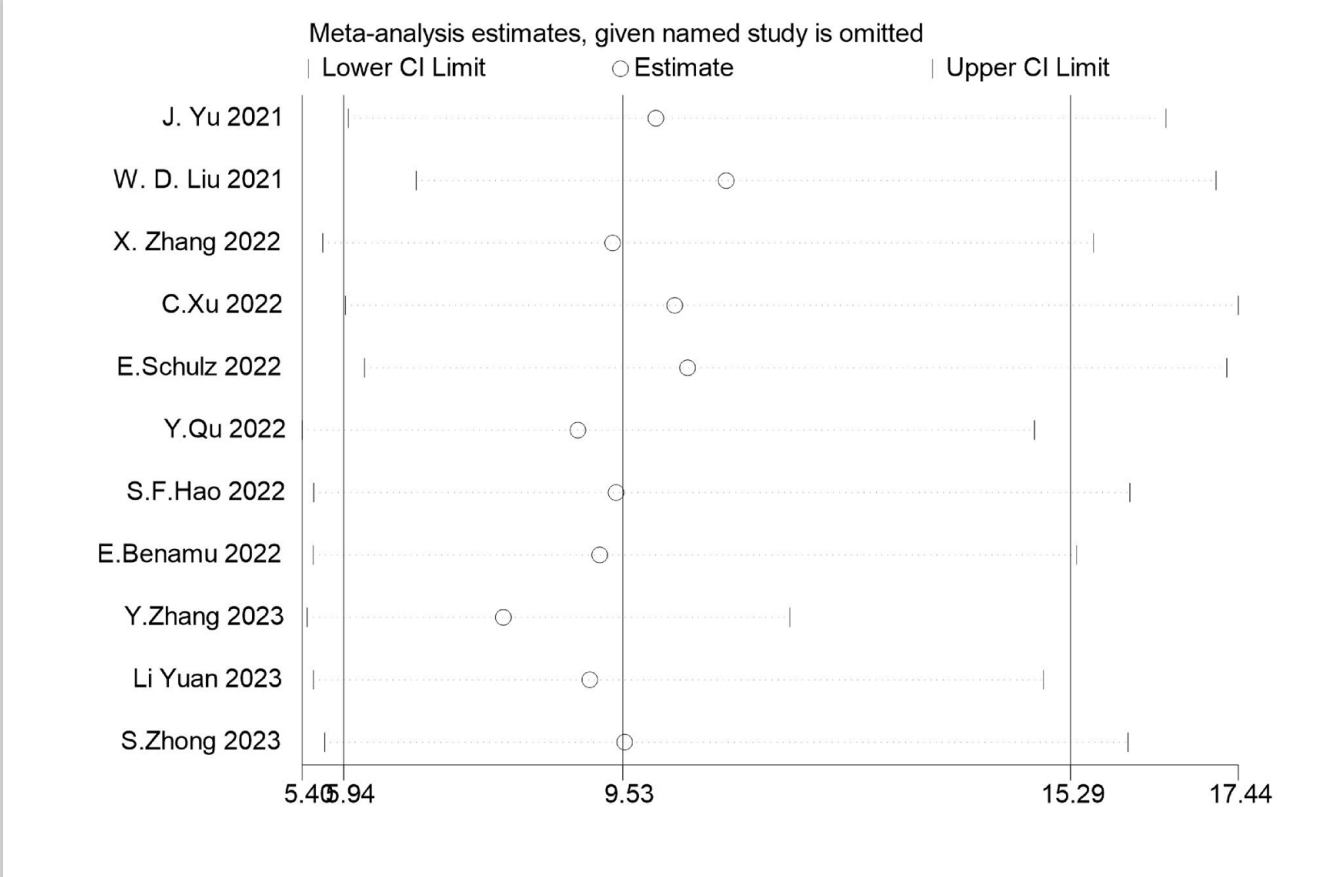


Figure S 3 Sensitivity analysis of BALF specimens


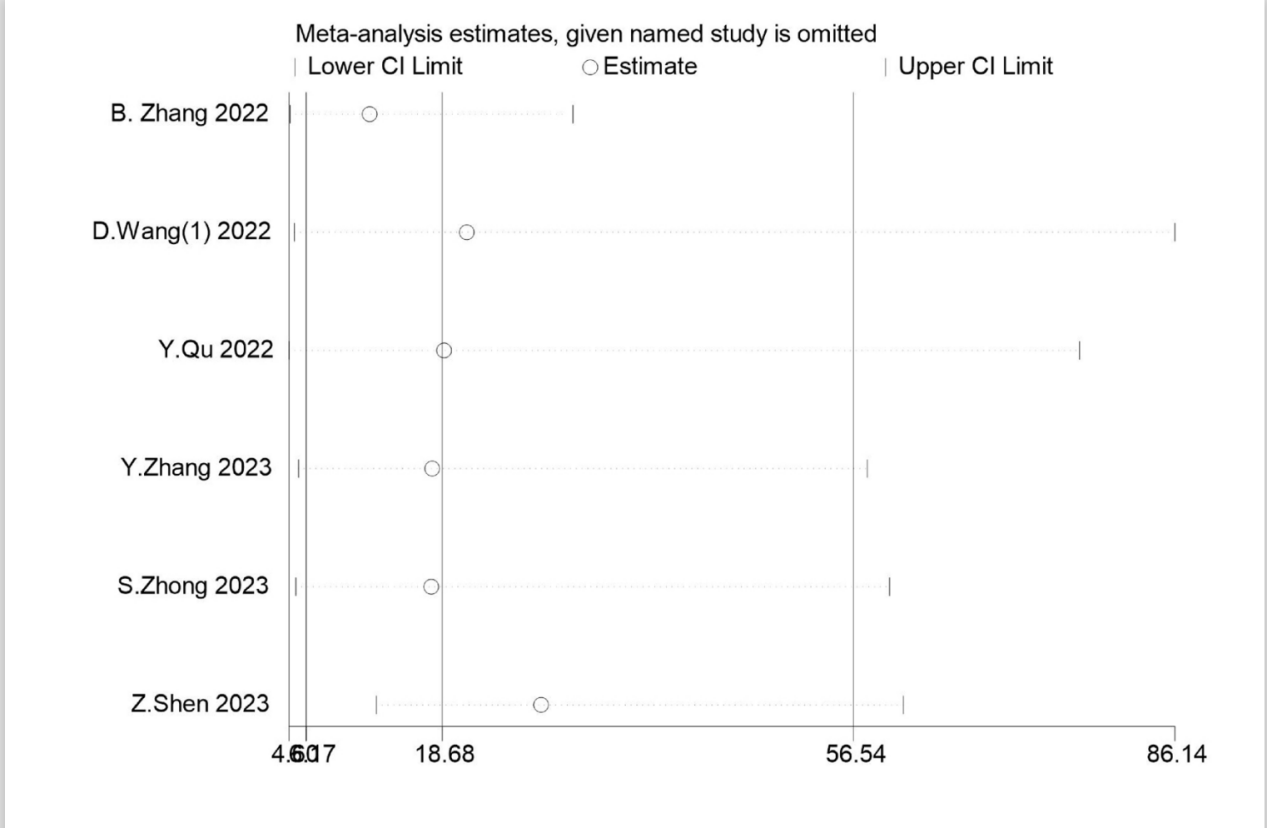


Figure S 4 Sensitivity analysis of other specimens


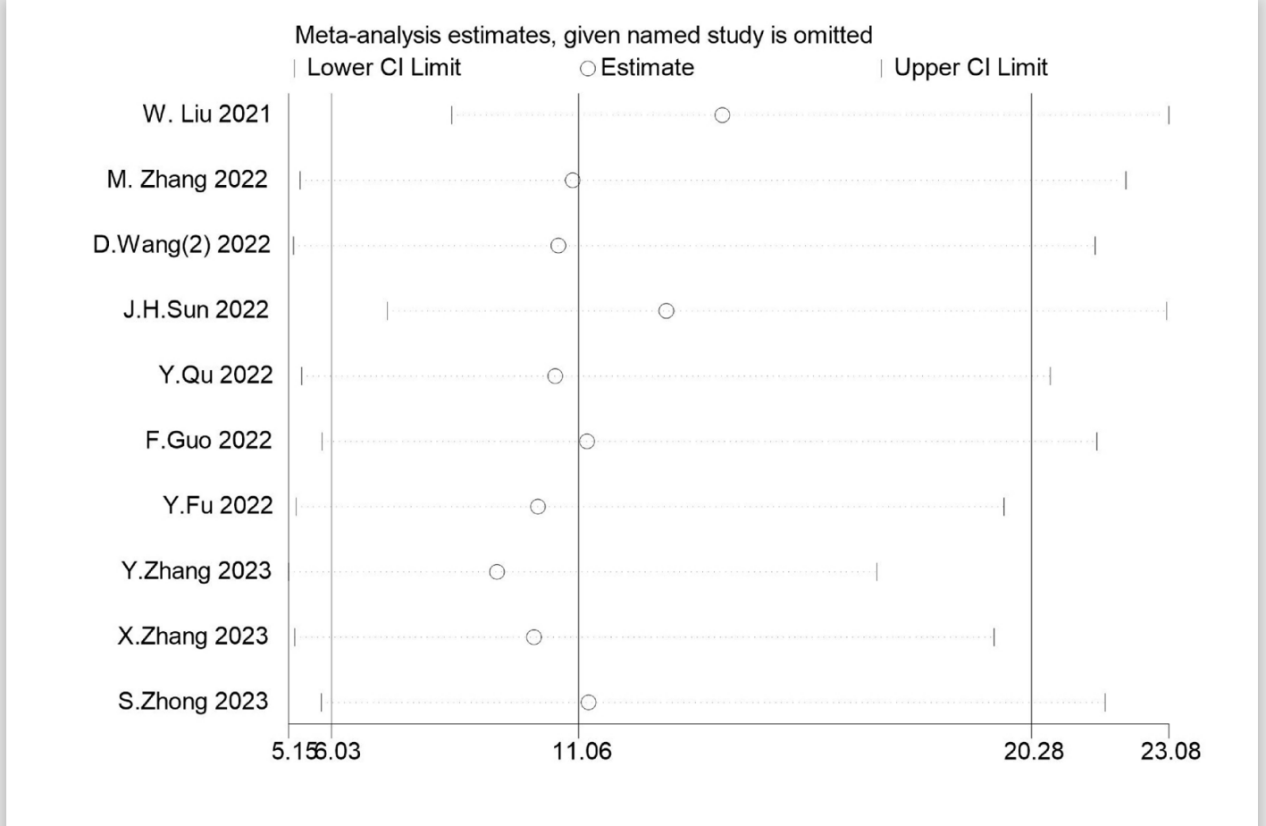


Figure S 5 Funnel plot of detection positive rate of different samples between mNGS and conventional methods group


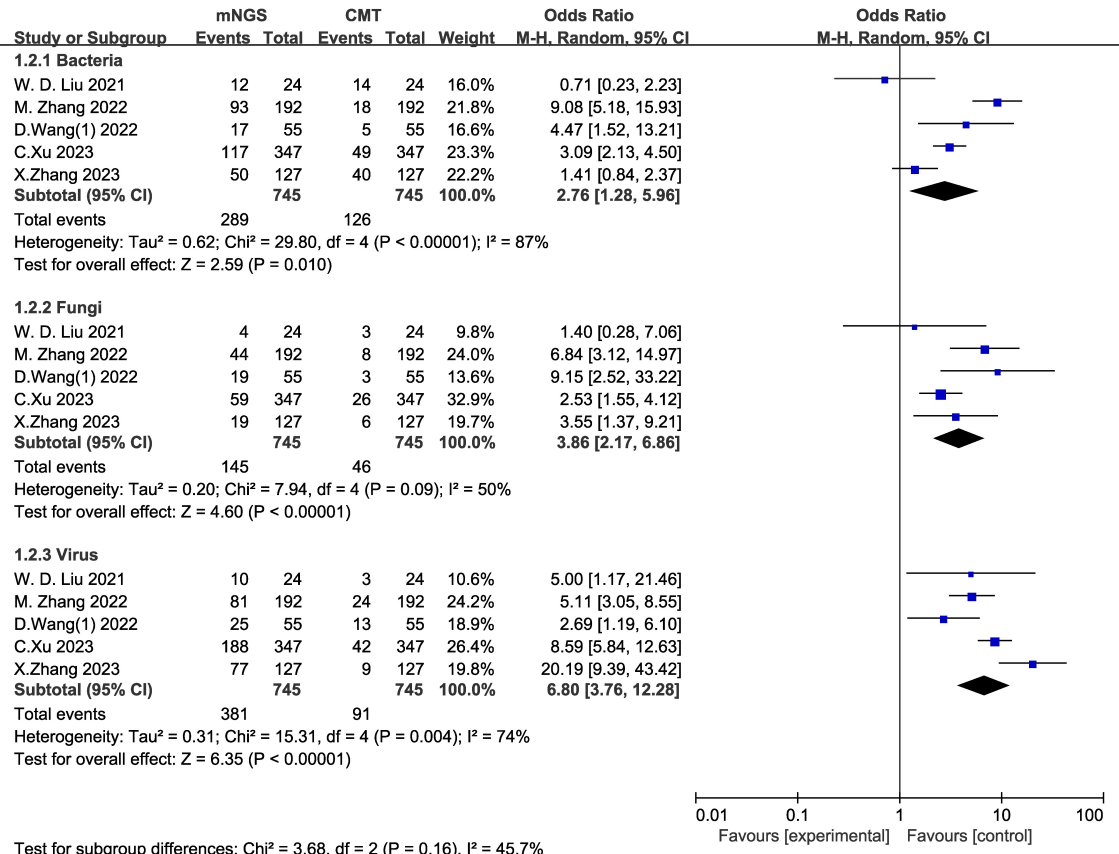


Figure S 6 Funnel plot of detection positive rate of different pathogens between mNGS and conventional methods group


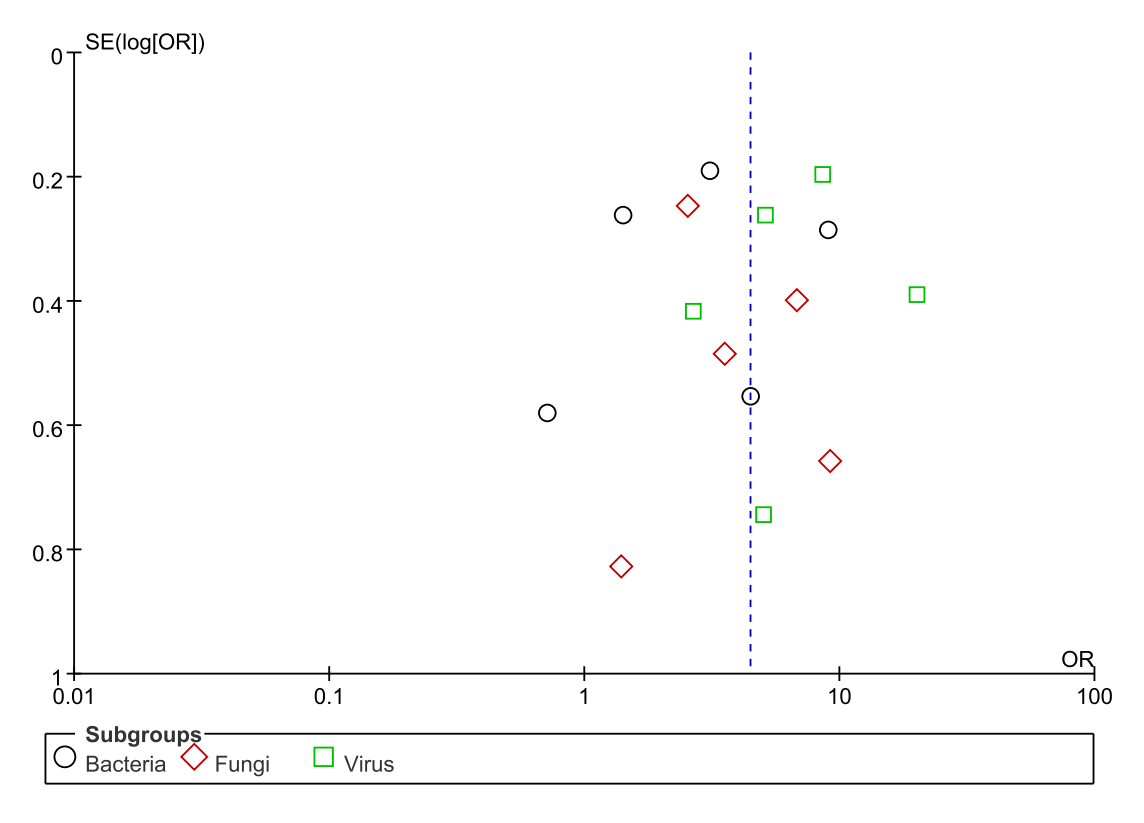


Figure S7 Comparison of detection positive rate of mNGS in the neutropenia group and non-neutropenia group


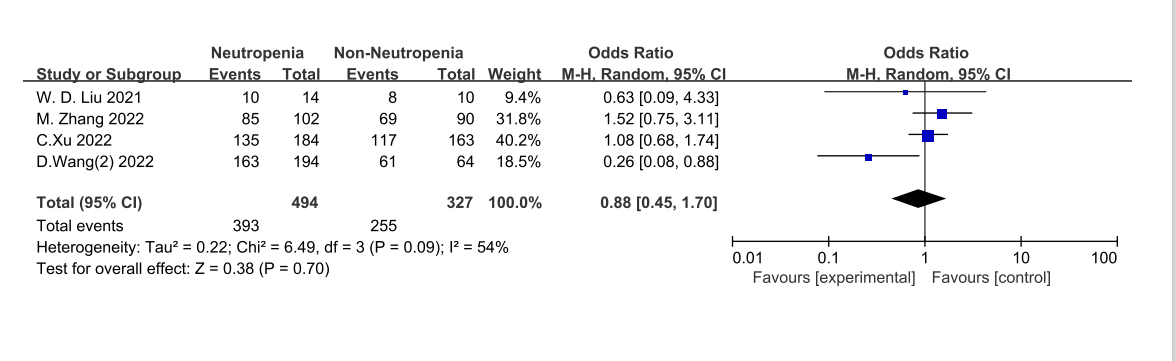


Figure S8 Forest plot for the sensitivity and specificity of mNGS for the diagnosis of infection in hematology patients when clinical diagnosis was used reference standard

**
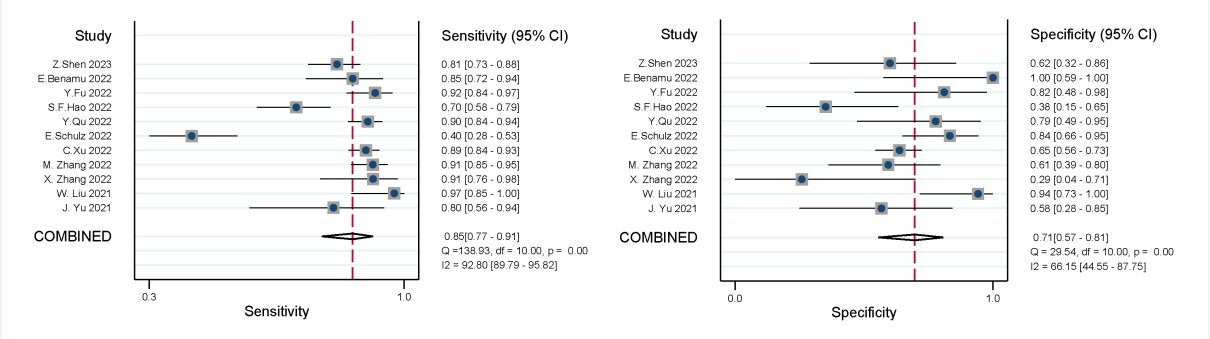
**

Figure S 9 Forest plot for the sensitivity and specificity of mNGS for the diagnosis of infection in hematology patients when CMT was used as reference standard

**
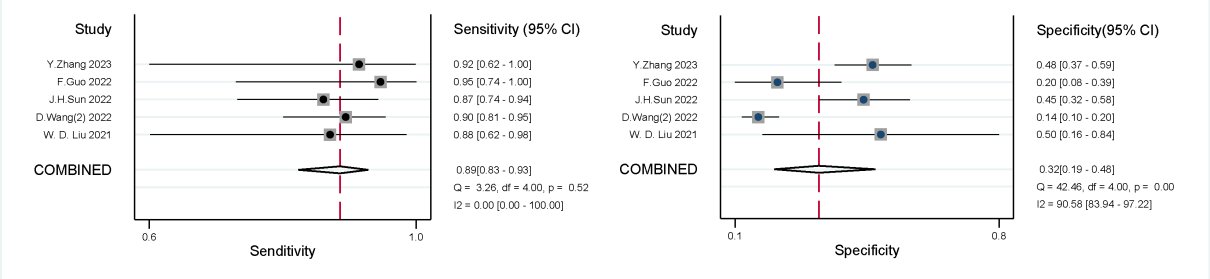
**
